# Biological Activity of Naturally Derived Naphthyridines

**DOI:** 10.3390/molecules26144324

**Published:** 2021-07-16

**Authors:** Gabriela Chabowska, Ewa Barg, Anna Wójcicka

**Affiliations:** 1 Department of Basic Medical Sciences, Faculty of Pharmacy, Wroclaw Medical University, Borowska 211A, 50-556 Wrocław, Poland; gabriela.chabowska@student.umed.wroc.pl; 2 Department of Pharmaceutical Technology, Faculty of Pharmacy, Wroclaw Medical University, Borowska 211A, 50-556 Wrocław, Poland

**Keywords:** natural compounds, alkaloids, naphthyridines, heterocyclic compounds, canthin-6-one, aaptamine, pyridoacridines, biological activity

## Abstract

Marine and terrestrial environments are rich sources of various bioactive substances, which have been used by humans since prehistoric times. Nowadays, due to advances in chemical sciences, new substances are still discovered, and their chemical structures and biological properties are constantly explored. Drugs obtained from natural sources are used commonly in medicine, particularly in cancer and infectious diseases treatment. Naphthyridines, isolated mainly from marine organisms and terrestrial plants, represent prominent examples of naturally derived agents. They are a class of heterocyclic compounds containing a fused system of two pyridine rings, possessing six isomers depending on the nitrogen atom’s location. In this review, biological activity of naphthyridines obtained from various natural sources was summarized. According to previous studies, the naphthyridine alkaloids displayed multiple activities, i.a., antiinfectious, anticancer, neurological, psychotropic, affecting cardiovascular system, and immune response. Their wide range of activity makes them a fascinating object of research with prospects for use in therapeutic purposes.

## 1. Introduction

The natural environment abounds in substances with multiple biological properties that have become an inspiration and basis for modern drugs. Since prehistoric times, secondary metabolites especially in the form of plant materials have been used for therapeutic purposes [1]. Nowadays, thanks to chemical sciences development, natural derivatives can be isolated from terrestrial and marine sources as multi-component extracts or single compounds. This provides a possibility to explore their properties, from chemical structures and general mechanisms of action to more specialized molecular targets.

**Figure 1:**
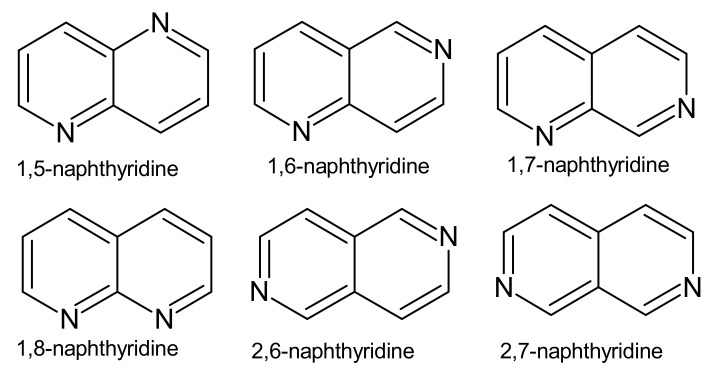
Isomeric forms of naphthyridines.

Naphthyridines are a class of heterocyclic compounds that are also referred to in the chemical literature as “benzodiazines” or “diazanaphthalenes”, due to possessing a fused system of two pyridine rings. There are six positional isomers with different locations of nitrogen atoms (Figure 1).

The compounds containing the naphthyridine scaffold are found in natural products (plants and marine organisms) or can be obtained synthetically. The properties and synthesis of 1,8-isomer derivatives were most often described, mainly due to nalidixic acid (1-ethyl-7-methyl-4-oxo-1,8-naphthyridine-3-carboxylic acid), which was discovered by G. Lesher in 1962 [2] and introduced into treatment in 1967 as an antibacterial drug. In the last several decades, scientists’ interest in naphthyridines has been growing due to their broad spectrum of biological activity. In this review, biological activity of naphthyridines derived exclusively from the natural environment is presented. Many of them revealed significant bioactivity and this article may encourage researchers to further the investigation of these chemical compounds.

## 2. Naturally Occurring Naphthyridine Derivatives

### 2.1. 1,5-Naphthyridine Derivatives

**Figure 2:**
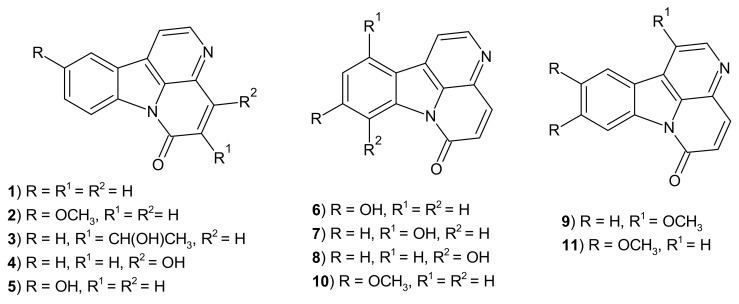
Structure of canthin-6-one derivatives.

1,5-Naphthyridine natural products are represented primarily by canthinone-type alkaloids. A major member of the group, canthin-6-one **1** (Figure 2), is isolated primarily from plants—the *Rutaceae* and *Simaroubaceae* families, but also from fungi [3]. The immunomodulatory activity of the compound has been determined. In rats with drug-induced colitis, canthin-6-one **1** reduced the production of pro-inflammatory mediators TNF-α (tumor necrosis factor α), IL-1β (interleukin-1β), IL-12p70 (interleukin-12p70), and VEGF (vascular endothelial growth factor). Moreover, it diminished oxidative stress in colon tissues [4].

Moreover, canthin-6-one **1** has been studied for its anticancer properties. Normally, cell death determines system homeostasis and prevents excessive proliferation and accumulation of defective cells. Major mechanisms of cell death comprise programmed apoptosis and autophagy, and traumatic necrosis. In cancer cells, cell death is disturbed due to genetic abnormalities. Restoration of a proper cell cycle together with generating cell damage in tumor tissues are key targets for anticancer compounds.

Canthin-6-one **1** was shown to activate apoptosis and necrosis in Kasumi-1human myeloid leukemia cells, with cell cycle arrest at G0/G1 and G2, respectively, at 7 μM and 45 μM. The agent also induced cancer cells differentiation, a process which could potentially lead to the conversion of neoplastic cells into normal [5].

1,5-Naphthyridine representatives were also obtained from *Zanthoxylum paracanthum Kokwaro*—an endemic, tropical plant, native to Kenya and Tanzania. Canthin-6-one and 10-methoxycanthin-6-one **2** (Figure 2), isolated from the species, have been regarded as promising antibacterial and antifungal substances [6]. They displayed strong inhibitory activity against *Staphylococcus aureus* and *Escherichia coli* (MIC values respectively of 0.49 and 3.91 µg/mL), and importantly also against methicillin-resistant *Staphylococcus aureus* strain (MIC values respectively 0.98, 3.91 µg/mL). The results of stronger canthin-6-one **1** were similar to that of reference omacilin. Antifungal effects of the compounds **1**–**2** were presented with MIC values, respectively, of 3.91 and 7.81 µg/mL for canthin-6-one **1** and 10-methoxycanthin-6-one **2**. The compounds **1**–**2** also exerted significant anticancer effects against DU145 prostate and HCC 1395 human breast cancer cell lines, with the most impressive activity of 10-methoxycanthin-6-one **2** against DU145 (IC_50_ = 1.58 µg/mL, and SI = 34.15) [6].

*Ailanthus altissima Swingle* has been considered as another natural source of 1,5-naphthyridines. It is a genus of tree distributed primarily in China, but is now widespread in Europe and North America. Healing properties of the plant are known in traditional medicine, and it is used i.a. in treating bacterial infections, fever and diarrheas. Kim et al. isolated six canthinone-type compounds, containing the 1,5-naphthyridine ring, from the bark of *Ailanthus altissima Swingle* [7]. The derivatives were determined as (*R*)-5-(1-hydroxyethyl)-canthine-6-one **3**, canthin-6-one **1**, 4-hydroxycanthin-6-one **4**, 10-hydroxycanthin-6-one **5**, 9-hydroxycanthin-6-one **6**, and 11-hydroxycanthin-6-one **7** (Figure 2). The compounds **1** and **3**–**7** were tested for their anti-inflammatory properties. Derivatives 1–5 showed strong inhibitory effect on LPS (lipopolysaccharides)-induced NO (nitric oxide) production in RAW 264.7 murine macrophage cell line (IC_50_ = 7.73–15.09 μM). Moreover, 10-hydroxycanthin-6-one showed antifungal activity against *Fusarium graminearum* and *Fusarium solani* (growth inhibition rates respectively of 74.5% and 57.9%), and antibacterial effect against *Bacillus cereus* (MIC = 15.62 µg/mL) [8]. Canthin-6-one displayed antiparasitic effect in mice infected with *Trypanosoma cruzi*, both in acute and chronic infection. Due to its low toxicity, it is considered a promising candidate in Chagas disease therapy [9]. Canthin-6-one and 8-hydroxy-canthin-6-one **8** were also shown to exert antimycobacterial effects [10].

1,5-Naphthyridine alkaloids were isolated also from *Leitneria floridana,* a species of shrub, commonly known as corkwood, which is distributed in the southern regions of the United States. *Leitneria floridana*-derived 1-methoxycanthin-6-one **9** appeared to exert an effect against HIV (Human Immunodeficiency Virus) with an EC_50_ value of 0.26 g/mL [11]. Beside antiviral properties, the compound displayed anticancer potency and was shown to induce cellular apoptosis by activation of c-Jun N-terminal kinase [12].

The protein complex NF-κB (nuclear factor kappa B) plays a key role in pro-inflammatory mechanisms and has been found chronically active in various types of tumors and autoimmune diseases. A study performed by Tran et al. showed that *Eurycoma longifolia*-derived alkaloids: 9-hydroxycanthin-6-one **6**, 9-methoxycanthin-6-one **10** and 9,10-dimethoxycanthin-6-one **11** (Figure 2) significantly inhibited NF-κB transcription with IC_50_ values in the range of 3.8–19.5 μM [13]. In another study, *Brucea mollis-*isolated 9-methoxycanthin-6-one **10** exerted strong cytotoxic properties against KB epidermoid carcinoma, LU-1 lung adenocarcinoma, LNCaP prostate adenocarcinoma, and HL-60 leukemia human cell lines with IC_50_ values in the range of 0.91–3.73 μM [14].

**Figure 3:**
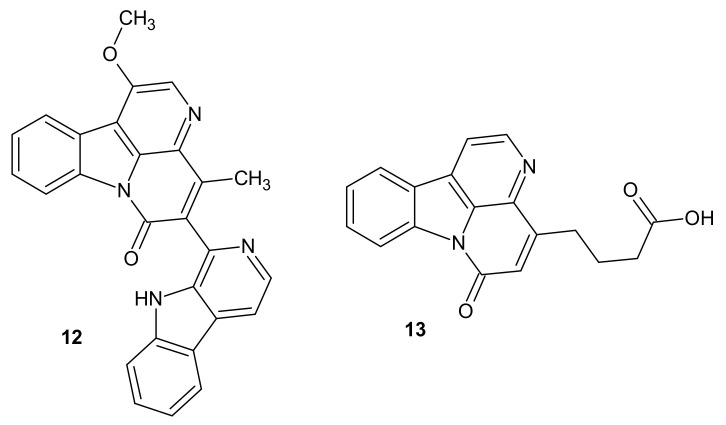
Structures of quassidine E **12** and canthin-16-one-14-butyric acid **13**.

*Picrasma quassioides,* a genus of tree commonly growing in temperate regions of southern Asia, revealed to be the source of another natural naphthyridine agents. Jiao et al. isolated novel 1,5-naphthyridine alkaloids from *Picrasma quassioides Bennet* [15]. The compounds were determined as quassidine E **12** and canthin-16-one-14-butyric acid **13** (Figure 3). The novel agents **12** and **13** reduced the production of pro-inflammatory mediators: NO, IL-6, and TNF-α in LPS-induced RAW 264.7 cells, with IC_50_ values in the range of 20.51–66.96 μM [15].

**Figure 4:**
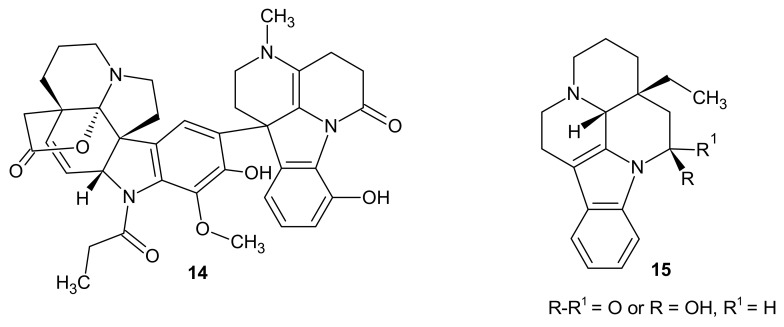
Structures of cimiciduphytine **14** and eburnane derivatives **15**.

Natural products—cimiciduphytine **14** and eburnane derivatives **15** (Figure 4)—were evaluated as natural painkillers and antihypertensive agents, which could be used in cerebral circulation disturbance therapy [16].

### 2.2. 1,6-Naphthyridine Derivatives

*Aaptos*, a widely-known genus of marine sponges, is considered as a prominent natural source of 1,6-naphthyridines. *Aaptos* was firstly described by Gray in 1867 [17]. The genus is represented by nearly 29 species that can be found in shallow waters of coastal areas all over the world. Since the 1980s, *Aaptos* has been extensively researched due to being the source of at least 62 secondary metabolites with diverse biological activities [18].

**Figure 5:**
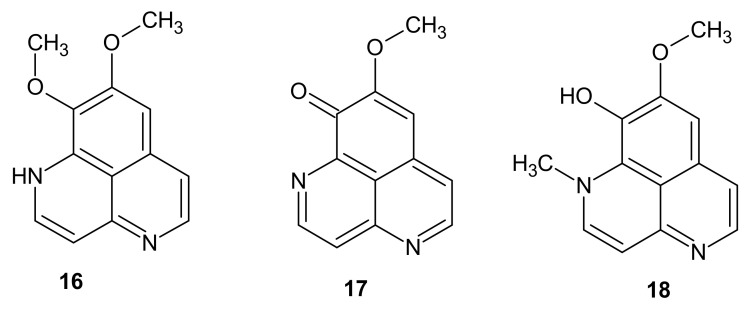
Aaptamine derivatives **16**–**18**.

Aaptamine (8,9-dimethoxy-1*H*-benzo[*de*][1,6]naphthyridine) **16** (Figure 5) isolated by Nakamura et al. [19] in 1982 from *Aaptos aaptos* became the first, maternity representative of aaptamines family. The anticancer activity of the compound **16** has been extensively researched. Aaptamine exhibited notable cytotoxic effects in vitro against H1299 and A549 non-small cell lung cancer [20], HeLa cervical cancer [21], and CEM-SS T-lymphoblastic leukemia cell lines [22], with IC_50_ values ranging from 10.47 to 15.03 μg/mL. Moreover, aaptamine **16** displayed a potent anticancer effect in mice carrying human hepatocellular carcinoma HCC-LM3 xenografts with downregulation of SOX9 and Ki67 expression [23]. The agent **16** has been detected to intercalate into DNA [24], upregulate p21 expression, and induce apoptosis in cancer cells in a p53-independent manner [23,25]. Aaptamine **16** expressed the ability to interfere specifically with p53 and c-myc network in NT2 human embryonal carcinoma cell line [26]. Gong et al. [20] performed further investigation of the mechanism of aaptamine **16** action in non-small cell lung cancer cell lines. The agent 16 displayed antiproliferative properties with inhibition of cancer cells growth and clonogenicity in a dose-dependent manner. Aaptamine induced G1 cell cycle arrest with a reduction of CDK2 (Cyclin-dependent kinase 2), CDK4 (Cyclin-dependent kinase 4), Cyclin D1, and Cyclin E levels, and also interfered with the PI3K/AKT/GSK3b (phosphatidylinositol-3 kinase/protein kinase B/glycogen synthase kinase 3 beta) axis. It could potentially diminish the process of metastasis and tumor invasion due to downregulation of MMP-7 (matrix metalloproteinase-7) and MMP-9 (matrix metalloproteinase-9) expression [20]. Beside anticancer activity, aaptamine **16** was shown to block α-adrenoceptors in vascular smooth muscles [27]. Hence, it could be considered a antihypertensive agent. Moreover, the compound **16** has been reported to display antiviral activity against HIV-1 [24] and anti-amoebic effect towards *Acanthamoeba castellanii* [28]. Aaptamine **16** selectively blocked the type A MAO (Monoamine Oxidase) [29], an enzyme which is overexpressed in the brain during major depression episodes. Therefore, aaptamine **16** could be useful in depression therapy. Aaptamine **16** demonstrated a wide range of activity, nevertheless its derivatives surpassed the effect of parental agent.

There are several studies comparing properties of parent aaptamine **16** with its derivatives demethyl(oxy)aaptamine **17** and isoaaptamine **18** (Figure 5). Dyshlovoy et al. [30] investigated anticancer properties of these compounds **16**–**18**, isolated from *Aaptos*. Evaluation of cytotoxicity on human cancer cell lines (THP-1 human leukemia monocytic, HeLa cervical cancer, SNU-C4 colorectal carcinoma, SK-MEL-28 human melanoma, MDA-MB-231 breast cancer) in MTS assay confirmed significant anticancer potency of aaptamines **16**–**18**, and aaptamine analogues exerted impressively higher activity than the parent compound. All the agents **16**–**18** induced apoptosis in THP-1 cell line, and the effect was also more significant for demethyl(oxy)aaptamine **17** and isoaaptamine **18**. The study confirmed p53-independent cell cycle arrest induced by these compounds **16**–**18**. The agents **16**–**18** were also shown to prevent cancerogenesis induced by epidermal growth factor at low, non-toxic concentrations in the JB6 P+ Cl41 murine epidermal cell line. The mechanism of prevention is independent from the transcription of AP-1 (activator protein-1) and NF-ϰB.

Wu et al. [31] investigated the cytotoxicity of aaptamine **16**, demethyl(oxy)aaptamine **17,** and isoaaptamine **18** on breast cancer cell lines. The derivatives **17** and **18** also presented higher activity than parent compound **16**. Isoaaptamine **18** exerted the highest effect on T-47D (IC_50_ = 30.13 µM), meanwhile demethyl(oxy)aaptamine **17** was the most potent on MCF-7 (IC_50_ = 23.11 µM) and MDA-MB-231 (IC_50_ = 19.34 µM) cell lines. Isoaaptamine **18**, chosen for further research as the most prominent alkaloid (84.74%) in the active fraction isolated from sponge *Aaptos* sp., presented short-term and long-term antiproliferative properties. The compound **18** inhibited XIAP (X-linked inhibitor of apoptosis protein) expression, and due to caspases 3 and 7 activation and cleavage of PARP (Poly ADP-ribose polymerase), induced apoptotic cell death in cancer cells. Cytotoxic properties of isoaaptamine **18** have also been based on autophagy induction, disruption to mitochondrial function, and over-generating reactive oxygen species. The role of isoaaptamine **18** as a significant apoptosis inducer on THP-1 cells was confirmed by Shubina et al. [32]. The compound **18** exerted the highest activity among tested aaptamine analogues.

**Figure 6:**
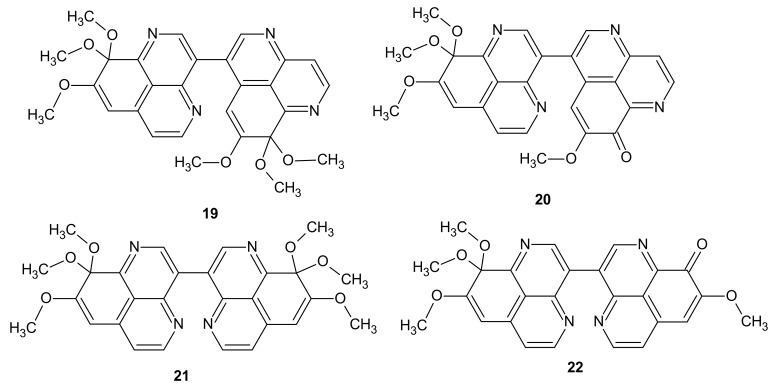
Structures of suberitines A-D **19**–**22**.

**Figure 7:**
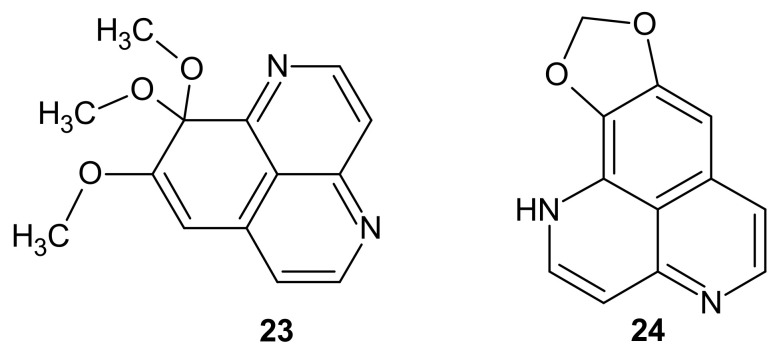
Structure of 8,9,9-trimethoxy-9*H*-benzo[*de*][1,6]naphthyridine 23 and 1,3-dioxolo[4,5-*d*]benzo[*de*][1,6]naphthyridine **24**.

**Figure 8:**
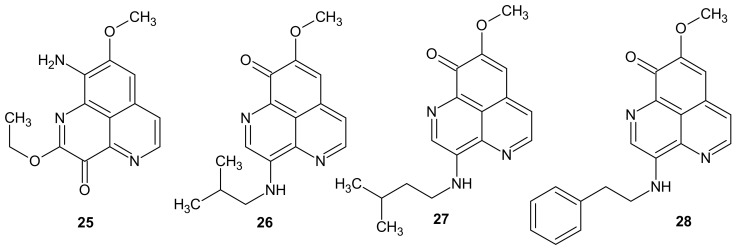
Structures of aaptamine derivatives **25**–**28**.

**Figure 9:**
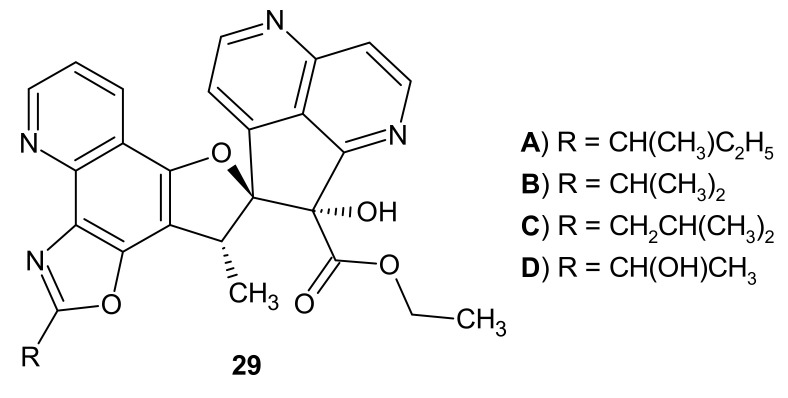
Structures of aaptodine A-D **29**.

Beside aaptamine **16** and its major analogues demethyl(oxy)aaptamine **17** and isoaaptamine **18**, other 1,6-naphthyridines have been successfully isolated from marine sponge *Aaptos.* Liu et al. [33] obtained four novel 1,6-naphthyridine alkaloids, suberitine A-D **19**–**22** (Figure 6), and two known alkaloids, demethyl(oxy)aaptamine **17** (Figure 5) and 8,9,9-trimethoxy-9*H*-benzo[*de*][1,6]naphthyridine **23** (Figure 7), from the *Aaptos suberitoides*. 

Cytotoxic evaluation of the compounds revealed significant antitumor activity of **20** and **22** against P388 cell line with IC_50_ values, respectively, of 1.8 and 3.5 μM [33].

Toshiyuki Hamada et al. [34] isolated 8,9,9-trimethoxy-9*H*-benzo[*de*][1,6]naphthyridine 23 and 1,3-dioxolo[4,5-*d*]benzo[*de*][1,6]naphthyridine **24** (Figure 7) from the Bornean *Aaptos aaptos* and tested their cytotoxicity against adult T-cell leukemia cells. Compound **24** revealed a significant antitumor effect with an IC_50_ value of 0.29 µM, while alkaloid **23** remained inactive.

Yu et al. [35] isolated nine novel and three previously-known aaptamine derivatives from the South China Sea sponge *Aaptos aaptos*. Four of the compounds: 9-Amino-2-ethoxy-8-methoxy-3*H*-benzo[*de*][1,6]naphthyridin-3-one 25, 3-isobutylaminodemethyl(oxy)aaptamine **26**, 3-(isopentylamino)demethyl(oxy)aaptamine **27**, and 3-(phenethylamino)demethyl(oxy)aaptamine **28** (Figure 8) revealed potent anticancer activity with IC_50_ values in the range of 0.03–8.5 μM against human cancer cell lines: HL60 leukemia, K562 erythroleukemia, MCF-7 breast cancer, KB epidermoid carcinoma, HepG2 hepatocellular carcinoma, and HT-29 colon adenocarcinoma [35].

RANKL (Receptor Activator for Nuclear Factor κB Ligand) is a transmembrane protein that controls bone regeneration and remodeling. Wang et al. [36] performed biological examination of four 1,6-naphthyridines analogues, aaptodine A–D **29** (Figure 9), derived from *Aaptos suberitoide*. Aaptodine A–D **29** inhibited impressively RANKL-induced osteoclast formation and resorption, with the strongest effect for aaptodine D **29D**. Hence, aaptodines could be considered as candidates for drugs used in treating osteoporosis and hormone therapy-induced bone loss.

Another rich source of 1,6-naphthyridine is constituted by *Sophora* derivatives. Trees and shrubs from genus *Sophora,* comprising approximately 62 species, are distributed in tropical and temperate zones in Eastern Europe*;* Asia; Australia; Pacific islands; and western, North, and South America. Natural alkaloids have been isolated from roots, seeds and epigeal parts of the plant. *Sophora* extracts have been used in traditional Chinese medicine [37].

**Figure 10:**
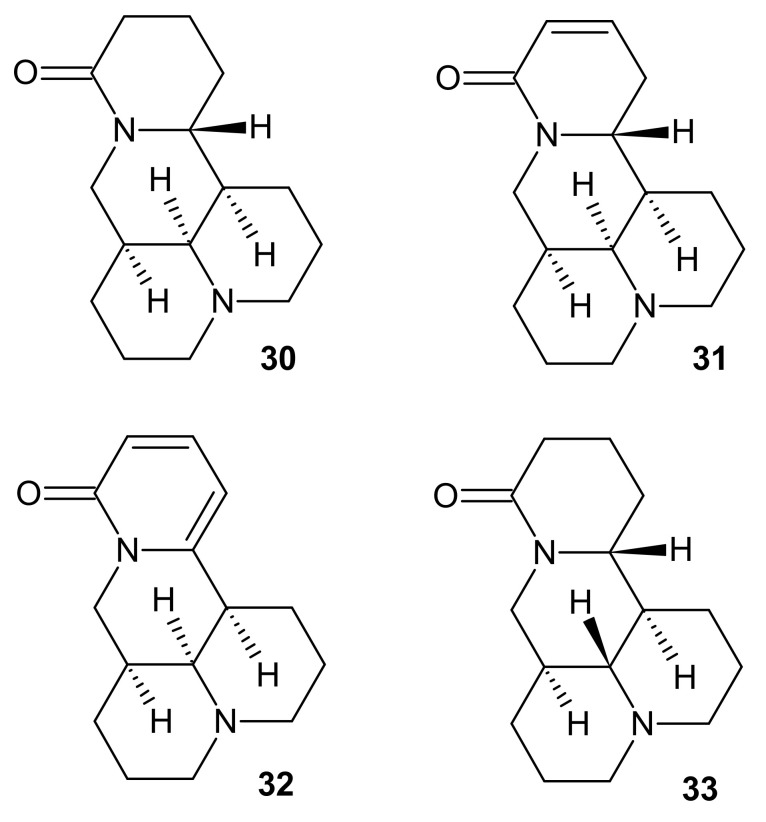
Structures of the Sophora alkaloids **30**–**33**.

Matrine **30** (Figure 10) is considered as one of the most principal and widely studied *Sophora* alkaloid, and the number of publications regarding the compound are constantly growing. Molecular mechanisms of matrine **30** antitumor activity have been considerably researched. Recent studies confirmed the antineoplasm effect of matrine on MCF-7 breast cancer and A549 non-small cell lung cancer cell lines by inhibiting AKT/mTOR axis [38,39]. Additionally, it reduced tumor growth of ovarian cancer cells in vivo by inducing the expression of ERK and JNK (c-Jun N-terminal kinase) pathways [40]. Matrine **30** also exerted a significant effect in drug-resistant tumors by inducing apoptosis and inhibiting efflux-pump activity [41,42]. Beside anticancer properties, matrine **30** exerted cardioprotective effects towards cardiomyocyte damage during hyperglycemia and sepsis [43,44]. Moreover, matrine **30** properties of protecting liver function resulted in several clinical trials. Intramuscular injections of matrine **30** caused improvement of condition in patients treated for chronic Hepatitis type B and patients suffering from primary hepatic carcinoma after trans-artery chemo-embolization [45,46]. Compound **30** was also reported to decrease total bilirubin level and improve survival rates in liver transplant recipients [47]. Matrine **30** is also considered a promising candidate as an immunosuppressive drug due to inhibiting autoimmune response in experimental models of multiple sclerosis [48,49]. It also exerts antiviral activity [50,51].

Extract isolated from the seeds of *Sophora alopecuroides* matrine **30,** sophocarpine **31** and sophoramine **32** (Figure 10), with contents, respectively, of 32.85%, 26.55%, and 6.91%, have alleviated morphine withdrawal in patients [52]. Moreover, these alkaloids **30**–**33** displayed strong antitumor properties. Matrine **30**, sophoridine **33** (Figure 10), and sophocarpine **31** exerted potent cytotoxic activity against HL-60 human leukemia, U937 human myeloid leukemia, K562 human erythroleukemia, EC109 esophageal squamous cell carcinoma, A549 non-small cell lung cancer, and HepG2 hepatocellular carcinoma cell lines, with IC_50_ values in the range of 1.21–12.86 mM [53]. Matrine **30** and sophocarpine **31** decreased cachexia symptoms (induced by colon carcinoma) in mice, and the mechanism of action was related to the suppression of TNF-α and IL-6 production [54]. Additionally, sophoramine **32** and sophocarpine **31** displayed antinematicidal activity against *Bursaphelenchus xylophilus*, pine trees parasite, and it was observed that degree of unsaturation in the -lactam ring correlated with the strength of antinematicidal activity [55].

**Figure 11:**
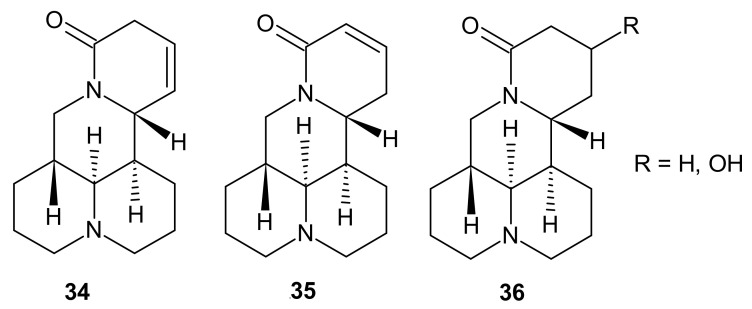
Sophoridine derivatives **34**–**36**.

*Sophora* alkaloids have been tested worldwide for their anti-inflammatory properties. Tang et al. [56] evaluated the activity of five 1,6-naphthyridines analogues **34**–**36** (Figure 11), derived from roots of *Sophora tonkinesis*. The agents **34**–**36** significantly reduced the secretion of cytokines TNF-α and IL-6 in LPS-stimulated murine macrophages RAW 264.7. The most potent effect, higher than that of matrine **30**, was exerted by 12,13-dehydrosophoridine **34** (TNF-α and IL-6 levels were 56.82% and 65.21%, respectively) [56].

**Figure 12:**
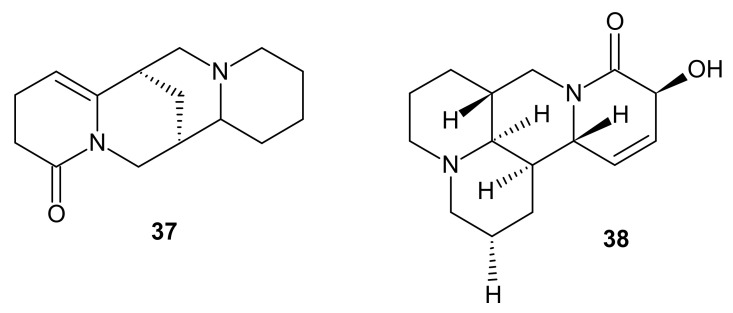
Structures of 5,6-dehydrolupanine **37** and sophalode K **38**.

Li et al. [57] isolated 43 natural alkaloids from the seeds of *Sophora alopecuroides*. Assessment of anti-inflammatory efficacy was measured as suppression of NO production in LPS-activated RAW 264.7 cells. Among *Sophora* alkaloids, 5,6-dehydrolupanine **37** (Figure 12) exerted the most potent effect (IC_50_ value of 25.86 μM), higher than that of matrine. On the other hand, another 1,6-naphthrydidine derivative sophalode K **38** (Figure 12) decreased significantly the secretion of enzymes playing crucial role in development of inflammation: iNOS (inducible nitric oxide synthase) and COX-2 (cyclooxygenase 2).

**Figure 13:**
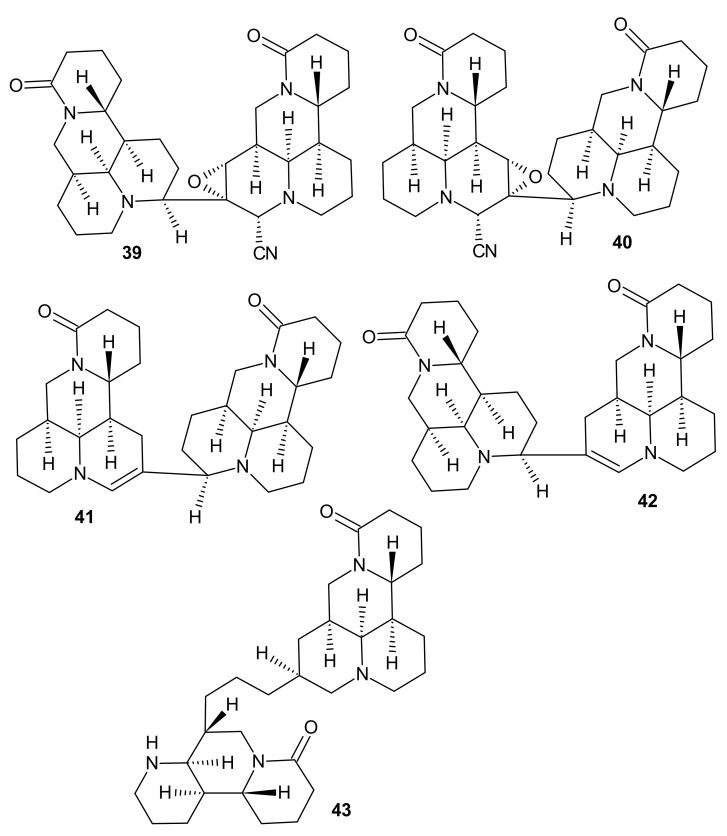
Alopecuroides A−E **39**–**43**.

Fan et al. [58] obtained five novel 1,6-naphthyridine alkaloid dimers, alopecuroides A−E **39**–**43** (Figure 13), from the aerial parts of *Sophora alopecuroides.* The study revealed strong anti-inflammatory properties of alopecuroides B **40** and C **41**. TNF-*α* and IL-6 levels were, respectively, 50.05% and 52.87% for alopecuroide B **35b** and 49.59% and 73.90% for alopecuroide C **41** in LPS-induced RAW 264.7 cells [58].

**Figure 14:**
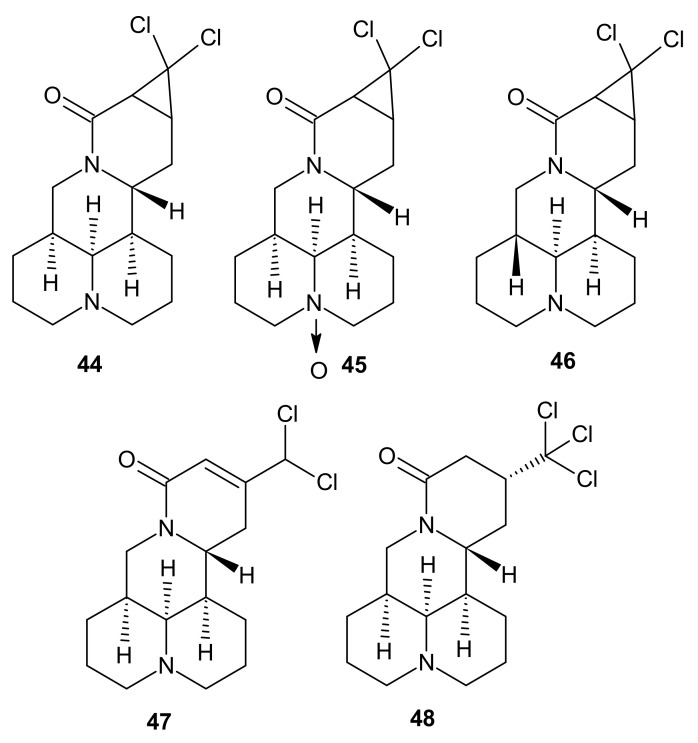
Alopecines A-E **44**–**48**.

*Sophora* alkaloids were also reported to exhibit immunosuppressive properties. Alopecines A-E **44**–**48** (Figure 14), isolated from the seeds of *Sophora alopecuroides,* were assessed toward inhibiting proliferation of Concanavalin A-induced T lymphocytes and LPS-induced B cells [59]. Alopecine D **47** exerted the most potent effect, with IC_50_ = 3.98 μM for inhibiting proliferation of T lymphocytes and 3.74 μM for B lymphocytes (SI ratios respectively 8.0 and 8.5).

**Figure 15:**
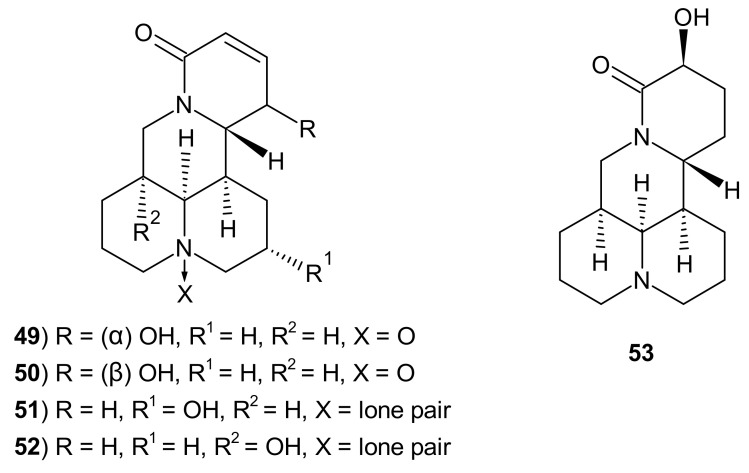
Structures of 12-hydroxyoxysophocarpine **49**–**50**, 9α-hydroxysophocarpine **51**, sophoranol **52,** and 14β-hydroxymatrine **53**.

Pan et al. [60] evaluated antiviral activity of 16 natural alkaloids, obtained from rhizomes of *Sophora tonkinensis*. 12α-Hydroxysophocarpine **49** (Figure 15), 12β-hydroxysophocarpine **50,** and sophoranol **52** inhibited Influenza Virus A/Hanfang/359/95 replication with IC_50_ values in the range of 63.07–242.46 μM (SI ratios 3.1–5.7). 12β-Hydroxyoxysophocarpine **50**, 9α-hydroxysophocarpine **51**, sophoranol **52,** and 14β-hydroxymatrine **53** (Figure 15) exerted the most potent activity against Coxsackie Virus B3 with IC_50_ values in the range of 26.62–252.18 μM (SI ratios 3.0–6.8) [60].

### 2.3. 1,7-Naphthyridine Derivatives

**Figure 16:**
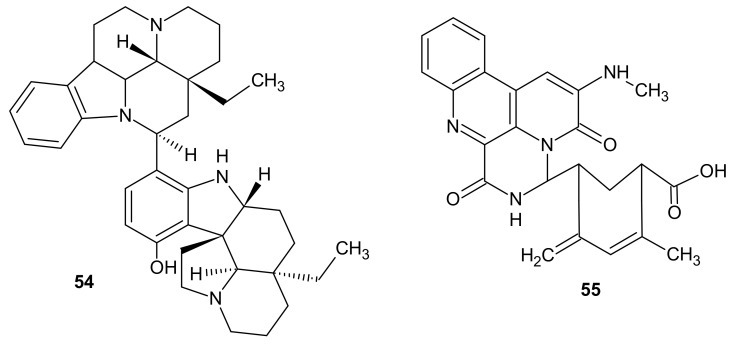
Bisleuconothine A **54** and 1-*N*-methyl-3-methylamino-[*N*-butanoicacid-3-(9-methyl-8-propen-7-one)-amide]-benzo[*f*][1,7]naphthyridine-2-one **55**.

Bisleuconothine A **54** (Figure 16), determined as 1,7-naphthyridine alkaloid, was derived from the bark of *Leuconotis* griffithii—a species of plant with native distribution in southern Asia [61]. The agent **54** was shown to exert antineoplasm potency by inhibiting WNT signaling pathway, and to induce G0/G1 cell cycle arrest in cancer cells. It displayed significant antiproliferative properties against SW480, HCT116, HT29, and SW620 colon cancer cells in vitro (IC_50_ values respectively 2.74, 3.18, 1.09, and 3.05 μM), and reduced tumor growth in mice carrying HCT116 Xenograft [62]. Research conducted by Wong et al. [63] in A549 non-small cell lung cancer and MCF-7 breast cancer cell lines showed that bisleuconothine A **54** ows its cytostatic properties to inducing autophagosome formation. Additionally, the compound **54** could also play a protective role in periodontitis. Bisleuconothine A **54** was determined to reduce RANKL expression and diminish periodontal tissue infiltration by pro-inflammatory polymorphonuclear cells [64].

*Streptomyces albogriseolus* is a mangrove bacterium species, widely known for producing aminoglycoside antibiotics neomycin B and neomycin C. *Streptomyces albogriseolus* has also been reported to be the source of 1,7-naphthyridine compound, 1-*N*-methyl-3-methylamino-[*N*-butanoic acid-3′-(9′-methyl-8′-propen-7′-one)-amide]-benzo[*f*][1,7]naphthyridine-2-one **55** (Figure 16) [65]. Cytotoxic evaluation performed by Tian et al. revealed that **55** displayed anticancer potency against HGC-27 human stomach carcinoma cell line [66].

### 2.4. 2,6-Naphthyridine Derivatives

**Figure 17:**
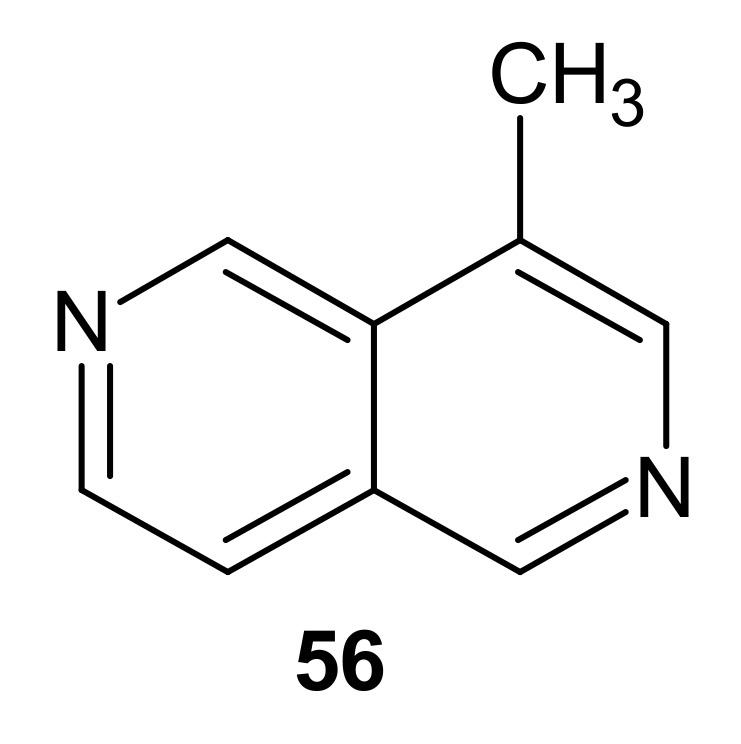
4-Methyl-2,6-naphthyridine **56**.

4-Methyl-2,6-naphthyridine **56** (Figure 17) is an alkaloid isolated from the dried plant of *Antirrhinum majus* by Harkiss and Swift in 1970 [67].

Some indolo[2,6]naphthyridine alkaloids were obtained from plants of *Erythrina* spp. The seeds of these tropical trees contain erythrina alkaloids of varying degrees of toxicity. Some of them are used by indigenous peoples for medicinal purposes. They have an effect on the central nervous system and exhibit hypnotic, curare-like effects and neuromuscular inhibition, as well as sedative and hypotensive activity [68,69].

**Figure 18:**
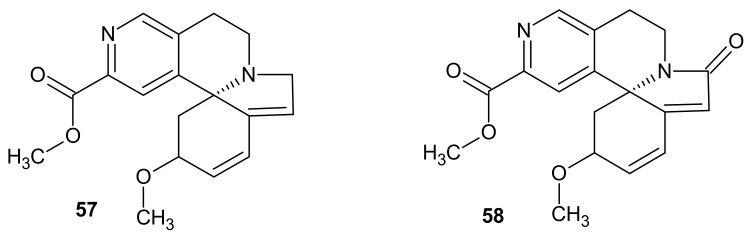
Erymelanthine **57** and melanacanthine **58**.

Erymelanthine-methyl(2*R*,13*bS*)-2-methoxy-2,6,8,9-tetrahydro-1*H*-indolo[7*a*,1*a*][2,6]naphthyridine-12-carboxylate **57** (Figure 18) was isolated from *Erythrina melanacantha* and *E. velutina* [70]. This alkaloid **57** was also obtained from *Erythrina merilliana* seeds by Jackson et al. [71]. Erymelanthine **57** was evaluated regarding its TRAIL (tumor necrosis factor-related apoptosis-inducing ligand) enhanced activity, and this alkaloid showed no cytotoxicity [72].

An example of Erythrina alkaloid containing indolo[2,6]naphthyridine scaffold is also 8-oxoerymelanthine 58, known as melanacanthine (methyl (2*R*,13*bS*)-2-methoxy-6-oxo-1,2,8,9-tetrahydroindolo[7*a*,1*a*][2,6]naphthyridine-12-carboxylate) **58** (Figure 18). This alkaloid **58** was isolated from *E. melanacantha* by Redha in 1983 [73]. Melanacanthine **58** inhibits thrombocyte agglutination and can be used in the treatment of hypertonia. The erythrina alkaloids **57**–**58** exhibited paralyzing activity [70,74].

**Figure 19:**
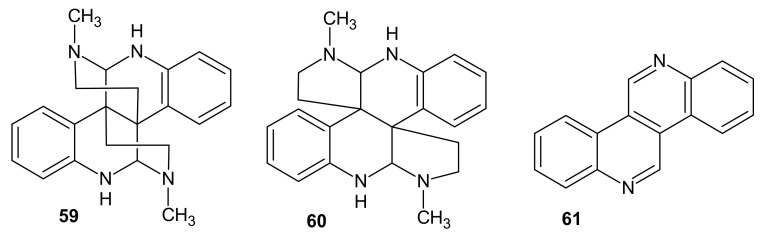
Calycanthine **59**, isocalycanthine **60,** and calycanine **61**.

Calycanthine **59** (Figure 19) was the first alkaloid isolated from the plants *Calycanthaceae* [75]. Calycanthine **59** was also isolated from *Meratia praecox* [76]. Toxic doses of calycanthine **59** cause excitation in mice, rats, and rabbits. After injection, calycanthine hydrochloride induces hyperglycaemia in rabbits and lowers blood pressure in cats or dogs [77]. Calycanthine **59** acts on peripheral motor nerves to cause muscle weakness associated in lower animals. In mammals, calycanthine causes seizures. The alkaloid **59** is also a strong depressant on the heart. It was reported that the anticonvulsant effect of calycanthine **59** is mainly caused by the inhibition of the release of the inhibitory neurotransmitter GABA (gamma-aminobutyric acid) [78].

Gordin [75] isolated a second alkaloid, isocalycanthine **60** (Figure 19), from the seeds of *Chimonanthus* genus. This tetradehydroisomer of calycanthine was also isolated from the leaves of *Psychotria colorata*, the plant used in folk medicine to relieve pain [79], and from *Psychotria forsteriana* [80].

Dibenzo[c,h][2,6]naphthyridine named calycanine **61** (Figure 19) was isolated from the seeds of *Chimonanthus praecox* [81]. This alkaloid **61** was also obtained by Zn dehydrogenation of calycanthine **59** [82]. Calycanthine **59** and calycanine **61** were evaluated for their antifungal activities. *Bipolaris maydis* was susceptible to calycanthine (EC_50_ = 29.3 μg/mL) [83]. Calycanthine **59** was evaluated for its potent melanogenesis inhibitory activity, but showed cytotoxicity at 10 µM [84].

### 2.5. 2,7-Naphthyridine Derivatives

Compounds containing the 2,7-naphthyridine scaffold were isolated from plants and various marine organisms. Naturally occurring bicyclic 2,7-naphthyridine derivatives are known as polycyclic compounds with the 2,7-naphthyridine ring in their structures.

#### 2.5.1. Bicyclic Alkaloids

**Figure 20:**
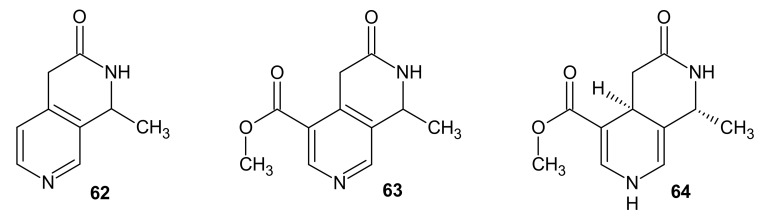
Jasminine **62**, jasminidine **63,** and dihydrojasminine **64**.

Some of the monoterpenoid alkaloids of 2,7-naphthyridine structure were isolated from *Oleaceae* species [85]. Jasminine **62** (Figure 20) was obtained from the leaf of *Ligustrum novoguineense* by Hart et al. [86]. Jasminine **62** at a dose of 300 mg/kg causes a slight decrease in motor activity in mice. Cardiovascular activity of this alkaloid **62** was also evaluated, but it showed no significant effects [87].

Jasminidine **63** was isolated from *Syringa Vulgaris* [88], and dihydrojasminine **64** (Figure 20) was isolated from *Osmanthus austrocaledonica* [89,90].

**Figure 21:**
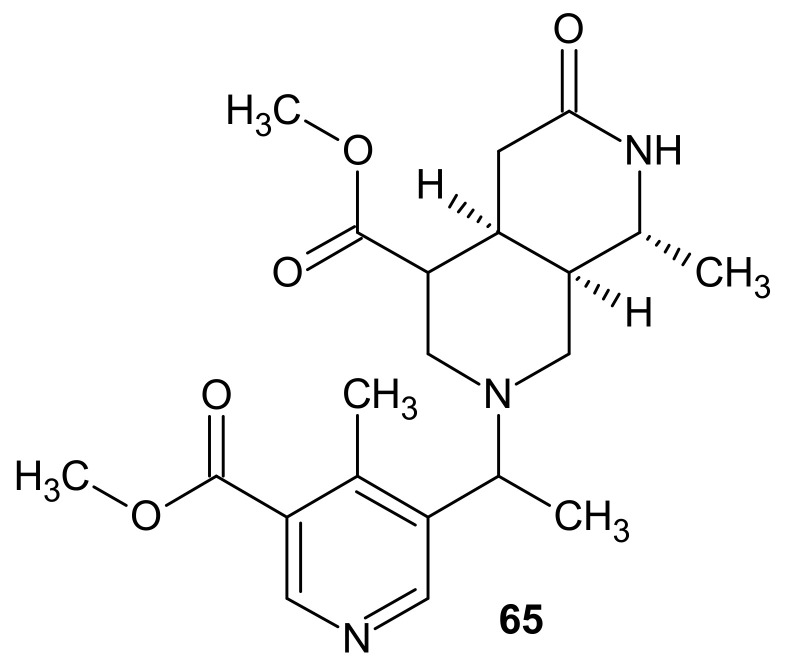
Austrodimerine **65**.

Benkrief et al. [89] also isolated jasminine **62**, dihydrojasminine **64,** and a new copyrine alkaloid—austrodimerine **65** (Figure 21) from *O*. *austrocaledonica*.

**Figure 22:**
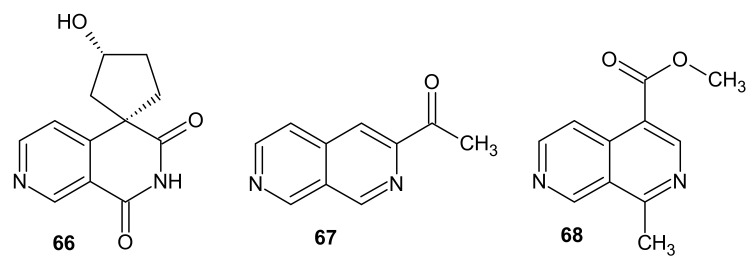
Sesbanine **66**, 3-acetyl-2,7-naphthyridine **67**, and neozeylancine **68**.

Powel et al. [91] reported antileukemic activity of extracts from seeds of *Sesbania drummondii*. Three years later, scientists described the isolation of alkaloid sesbanine—(3’*R*,4*R*)-3’-hydroxyspiro[2,7]naphthyridine-4,1’-cyclopentane-1,3-dione **66** (Figure 22) from the ethanol extract of *Sesbania drummondii* seeds, which are poisonous [92]. The extract containing sesbanine **66** showed cytotoxic activity (KB assay) and was active in vivo in the P-388 lymphocytic leukemia system [93].

3-Acetyl-2,7-naphthyridine **67** (Figure 22) was isolated by Janot et al. [94] from the roots and rhizomes of *Valeriana officinalis*. This compound **67** exhibited sedative and tranquilizer activity [94].

Neozeylancine **68** (Figure 22) was isolated in 1988 from *Neonauclea zeylanica* by Atta-ur-Rahman [95].

**Figure 23:**
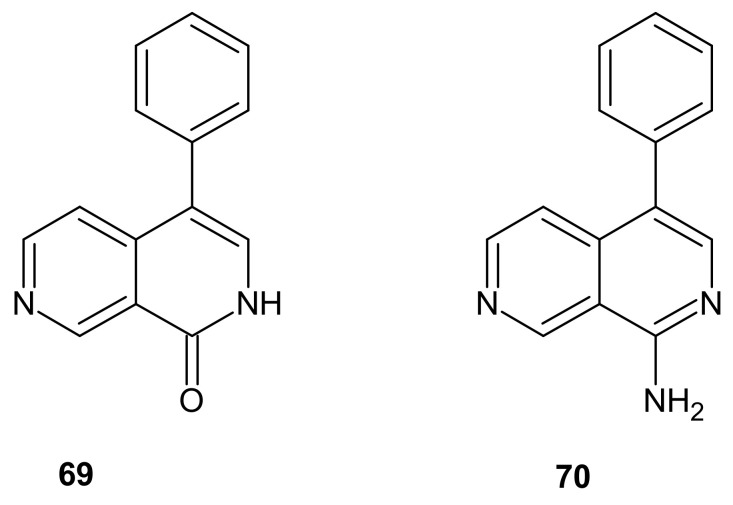
Structure of lophocladines **69**–**70**.

Bioactive alkaloids Lophocladine A **69** and lophocladine B **70** (Figure 23) were isolated from the marine red alga *Lophocladia* sp. by Gross et al. [96]. 4-Phenyl-2,7-naphthyridin-1-one **69** exhibited antagonistic activity against δ-opioid receptors, and 1-amino-4-phenyl-2,7-naphthyridine **70** showed cytotoxic activity against human lung tumor and breast cancer cell lines [96].

#### 2.5.2. Tricyclic Alkaloids

**Figure 24:**
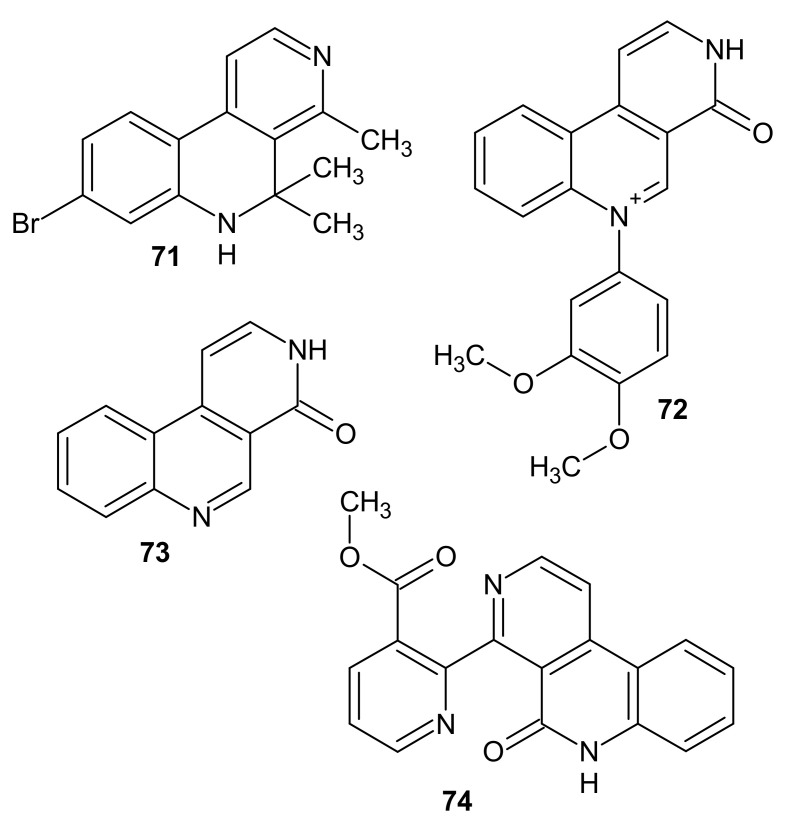
Structures of benzo[2,7]naphthyridine alkaloids **71**–**74**.

Some alkaloids containing a diazaphenanthrene (benzo[2,7]naphthyridine) scaffold were discovered in some plants and marine organisms. 8-Bromo-4,5,5-trimethyl-5,6-dihydrobenzo[*c*][2,7]naphthyridine named Veranamine **71** (Figure 24) was isolated from the ethanol extract of the marine sponge *Verongula rigida*. Veranamine **71** had a moderate affinity for serotonin receptors and was therefore assessed for antidepressant activity in mice using the forced swim test. This alkaloid **71** showed antianxiety and antidepressant activity and selective affinity for 5HT2B and sigma-1 receptors [97].

Alkaloids: 6-(3,4-dimethoxyphenyl)-3*H*-benzo[*f*][2,7]naphthyridin-6-ium-4-one called Perloline **72** and benzo[*f*][2,7]naphthyridin-4(3*H*)-one called perlolidine **73** (Figure 24) were isolated from the perennial rye grass *Lolium perenne* [98]. These alkaloids **72**–**73** inhibit in vitro cellulose digestion. Perloline **72** is slightly toxic after oral administration to mice and sheep [99].

Subarine-methyl 2-(5-oxo-6*H*-benzo[*f*][2,7]naphthyridin-4-yl)pyridine-3-carboxylate **74** (Figure 24) was isolated from Singaporean ascidian by Nilar et al. [100]. This marine alkaloid **74** was evaluated by in vitro screening against yeast and many Gram-positive and Gram-negative bacteria, but it exhibited no significant activity. Subarine **74** was also tested for in vitro cytotoxic activity on 60 human tumor cell lines, but did not show antiproliferative effect [101,102].

#### 2.5.3. Tetracyclic Alkaloids

**Figure 25:**
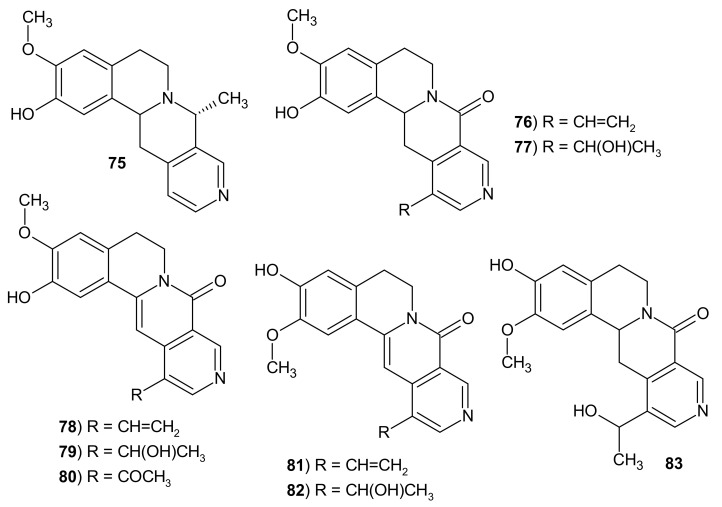
Structures of alangium alkaloids **75**–**83**.

The extract of the plant *Alangium lamarckii* has been used by Indians to treat many human disorders [103]. In Thailand, it is useful in the treatment of asthma, coughs, hemorrhoids, diarrhea; and in India to treat leprosy, fever, or as an anthelmintic agent [104,105]. Pakrashi et al. [106,107] isolated tetracyclic alkaloids from the seeds of *A. lamarckii*. This alangium alkaloids namely: alamaridine **75,** alangimaridine **76,** dihydroalamarine **77**, alangimarine **78**, alamarine **79**, alangimarinone **80**, isoalangimarine **81** isoalamarine **82,** and dihydroisoalamarine **83** (Figure 25) contain the isoquino[2,1-*b*][2,7]naphthyridine scaffold [108,109].

**Figure 26:**
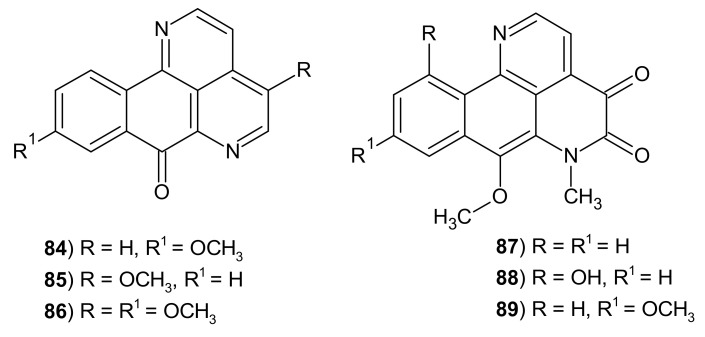
Eupomatidines 1–3 **84**–**86** and imbilines 1–3 **87**–**89**.

Some tetracyclic alkaloids possess 2,7-naphthyridine ring in the structure of azaaporphine: eupomatidines 1–3 **84**–**86** and imbilines 1–3 **87**–**89** (Figure 26) were isolated from *Eupomatia bennettii* and *E. laurina* [110].

Kitahara et al. [111] synthesized eupomatidines 1–3 **84**–**86** and evaluated their antifungal activity against *Candida albicans, Paecilomyces variotii*, and *Trichophyton mentagrophytes*. Eupomatidine-1 **84** exhibited activity against those tree fungi with EC_50_ values of 50 μg/mL, 6.25 μg/mL, and 0.4 μg/mL, respectively. Eupomatidine-2 **85** and eupomatidine-3 **86** were active only toward *T*. *mentagrophytes* with EC_50_ values 3.1 μg/mL and 6.25 μg/mL, respectively. Khan et al. [112] evaluated eupomatidine-1 **84** against over a dozen Gram-positive and Gram-negative bacteria strains, a protozoan and nine fungi. This alkaloid **84** turned out to be much better than the applied standard (ketoconazole and griseofulvin), both in terms of scope and level of antifungal activity. Eupomatidine-1 **84** showed comparable antimicrobial activity to chloramphenicol.

Imbiline-1 **87** (Figure 26) was also isolated from a large tree *Duguetia hadrantha* and then evaluated for its antimalarial and antimicrobial activity [113]. Imbiline-1 **87** showed a weak antimalarial potency, but it was more active against chloroquine-resistant than chloroquine-sensitive *Plasmodium falciparum* clones. Imbiline-1 **87** was found to be inactive against *C. albicans*, *C. neoformans,* and *S. aureus*. This alkaloid **87** exhibited cytotoxic activity in vitro against human malignant melanoma and human ovary carcinoma cell lines with IC_50_ values of 2 μg/mL and 5 μg/mL, respectively [113].

**Figure 27:**
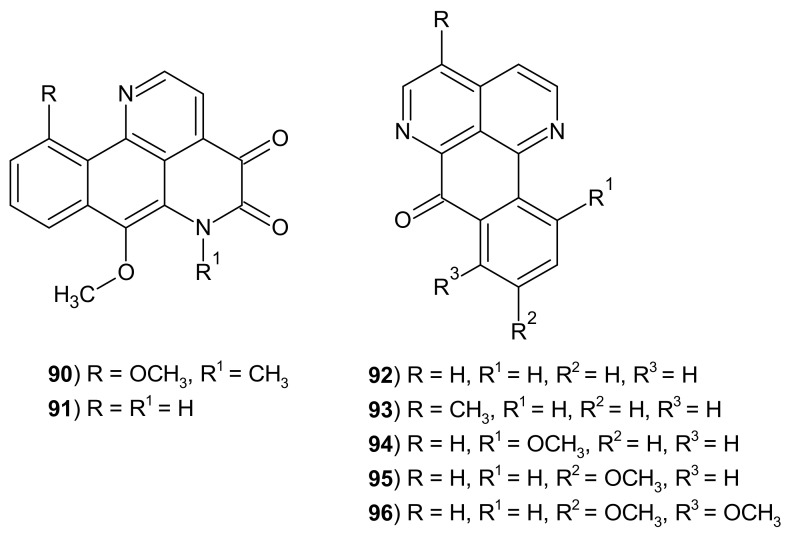
Hadrantines A **90** and hadranthine B **91, and** sampangines **92**–**96**.

The scientists [113] isolated also new imbiline-type alkaloids: hadranthine A **90a** and hadranthine B **90b** from the ethanolic extract of *Duguetia hadrantha* (Figure 27).

Hadranthine A (7,10-dimethoxy-6-methyl-4,5-dihydronaphthol[1,2,3-*ij*][2,7]naphthyridine-4,5-(6*H*)-dione) 90 showed in vitro antimalarial activity against chloroquine-resistant P. falciparum with IC50 = 120 ng/mL. This alkaloid was active against C. albicans with MIC = 20 μg/mL. Hadranthine B (7-methoxy-4,5-dihydronaphthol[1,2,3-*ij*][2,7]naphthyridine-4,5-(6*H*)-dione) **91** did not show antimalarial activity, but it exhibited in vitro cytotoxic activity against human malignant melanoma, epidermoid carcinoma, ductal carcinoma, and ovary carcinoma cell lines with IC_50_ values between 3–6 μg/mL [113]. This alkaloid **91** was also evaluated for its in vitro effects on immune response and inflammation, but did not show significant potency [113].

Sampangines **92**–**96** (Figure 27) also are tetracyclic alkaloids containing the 2,7-naphthyridine scaffold. Sampangine **92** was isolated first from the stem bark of plant *Cananga odorata* by Rao et al. [114]. 3-Methoxy derivative **93** was isolated from *Cleistopholis patens* by Liu et al. [115].

Sampangine **92** and 3-methoxysampangine **93** were also isolated from *Duguetia hadrantha* and then evaluated for their antimalarial, antifungal, and cytotoxic potency [115]. Both alkaloids **92**–**93** exhibited activity against *P. falciparum* with no cytotoxicity toward VERO cells. Sampangine **92** showed cytotoxicity to human malignant melanoma with IC_50_ = 0.37 μg/mL and inhibited cell aggregation (MIC < 0.15 μg/mL) [113]. Sampangine **92** isolated from the stem bark of *Anaxagorea dolichocarpa* by Lucio et al. [116] demonstrated antitumor activity against human leukemic strains with IC_50_ values of 10.15–11.80 μg/mL. Sampangine **92** and 11-methoxysampangine **94** isolated from the roots of *Ambavia gerrardii* were evaluated for their antiproliferative activity [117]. Alkaloids **92** and **94** showed strong activity against human non-small cell lung cancer cell line with IC_50_ values of 0.57–0.58 μM, but sampangine **92** was more active against human ovarian cancer cell line (IC_50_ = 0.60 μM) than its 11-methoxy derivative **94** (IC_50_ = 10.30 μM) [117]. Research by scientists under Kluza [118] directions has shown that sampangine **92** induces apoptosis in HL-60 cells. In low concentrations, it caused G1 arrest and at the same time induced mitochondrial hyperpolarization. At higher concentrations, it elicited mitochondrial depolarization [118]. The treatment of human leukemia cells with sampangine **92** (40 μM) induced apoptosis due to an oxidative stress [119]. The ability of sampangine **92** to produce reactive oxygen species was confirmed by using an in vitro biochemical assay, and the participation of this alkaloid **92** in binding and damaging DNA was excluded [120].

Sampangine **92**, and two derivatives: 9-methoxysampangine **95** and 8,9-dimethoxysampangine **96,** were isolated from the barks of *Polyalthia nemoralis* [121] and evaluated for their cytotoxic activity. Obtained alkaloids **92, 94, 95** exhibited significant cytotoxicity against human carcinoma in the mouth, human breast cancer, human lung cancer, human hepatoma cancer, human prostate cancer, human ovarian adenocarcinoma, and human colon adenocarcinoma [121].

Sampangines **92**–**93** exhibited antimicrobial activity against *C. albicans, C. neoformans,* and *S. aureus* [113,122]. Sampangine **92** was found to be a strong antifungal agent against *Paecilomyces variotii*, and *Trichophyton mentagrophytes* with EC_50_ = 0.2 μg/mL [111]. Scientists reported that the antifungal activity of sampangine **92** may be due to perturbations in heme biosynthesis or metabolism [123].

3-Methoxysampangine **93** showed significant in vitro antifungal activity against *C. albicans* and *C. neoformans* and *A. fumigatus,* better than amphotericin B [115]. These alkaloids **92**–**93** also showed excellent antimycobacterial activity against *Mycobacterium intracellulare*, comparable to rifampin [124]. The dependence of the activity of sampangine derivatives **92**–**96** on the type of substituents in the naphtho[1,2,3-*ij*][2,7]naphthyridine scaffold is presented in Table 1.

**Figure 28:**
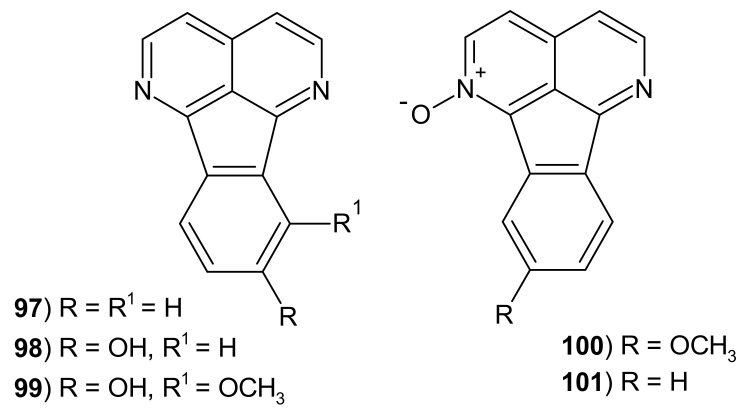
Structures of eupolauridine analogs **97**–**101**.

Eupolauridines are tetracyclic alkaloids containing the 2,7-naphthyridine scaffold condensed with an indene ring (Figure 28).

Eupolauridine (indeno[1,2,3-*ij*][2,7]naphthyridine) 97 was isolated from *Cananga odorata* [125] and from *Eupomatia laurina* [126]. Pan et al. [117] isolated eupolauridine **97**, 8-hydroxyeupolauridine **98**, 9-methoxyeupolauridine-1-oxide **100,** and eupolauridine *N*-oxide **101** from *Ambavia gerrardii*. Alkaloids were screened for their in vitro antiproliferative activity. Among eupolauridine analogues, compound **101** was the most active against human ovarian cancer cell line with IC_50_ = 3.5 μM, and only this derivative **101** showed antitumor activity against non-small-cell lung cancer cell line (IC_50_ = 1.77 μM) [117]. 7-Methoxy-8-hydroxyeupolauridine **99** isolated from *Polyalthia nemoralis* by Oanh et al. [121] did not show cytotoxicity against seven tested cancer cell lines (human carcinoma in the mouth, human breast cancer, human lung cancer, human hepatoma cancer, human prostate cancer, human ovarian adenocarcinoma, and human colon adenocarcinoma). Eupolauridine **97** extracted from the root bark of *Cleistopholis patens* by Hufford et al. [127] exhibited a significant activity against *C. albicans* with MIC = 1.56 μg/mL.

#### 2.5.4. Pentacyclic Alkaloids

**Figure 29:**
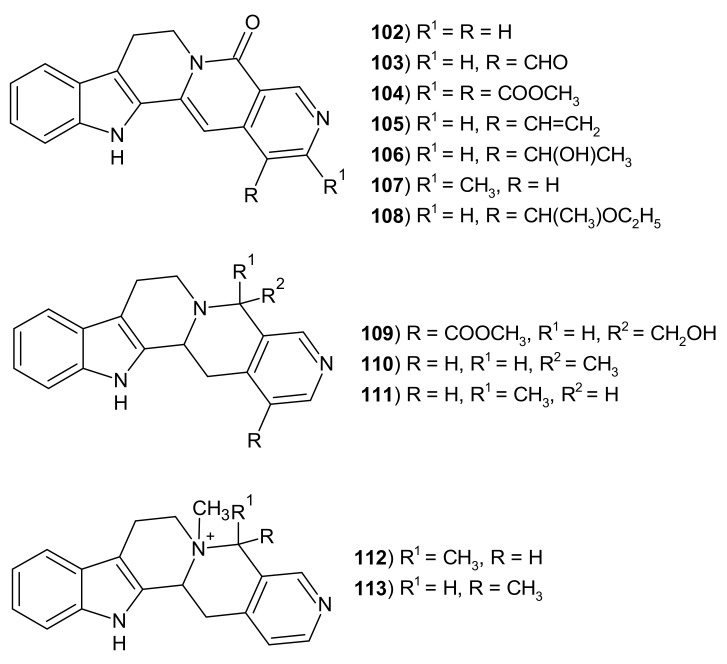
Structures of indolo[2′3′:3,4]pyrido[1,2-*b*][2,7]naphthyridine derivatives **102**–**113**.

Pentacyclic derivatives of indolo[2′3′:3,4]pyrido[1,2-*b*][2,7]naphthyridine (Figure 29) were isolated from different plants. Alkaloids: nauclefine **102**, naucletine **103**, angustine **105**, angustoline **106**, and angustidine **107** isolated from *Neuclea officinalis* [128], and neonaucline **104** and cadamine **109** isolated from *Ochreinauclea maingayii* exhibited potent vasorelaxant activity on isolated rat aorta [129]. Neuclefine **102** isolated from the bark of *Nauclea subdita* also induced apoptosis of diverse cancer cells and inhibited tumor xenograft growth [130]. Normalindine **111** and norisomalindine **110** were isolated from *Strychnos johnsonii* [131]. Isomalindine **113** and normalindine **111** were isolated from *Ophiorrhiza* sp. [132]. 19-*O*-ethylangustoline **99** and other alkaloids were isolated from the stem bark of *Sarcocephalus latifolius* [133]. Malindine **112** and isomalindine **113** were isolated from the stem bark of *Strychnos usambarensis* [134].

#### 2.5.5. Polycyclic Alkaloids—Pyridoacridine Analogs

**Figure 30:**
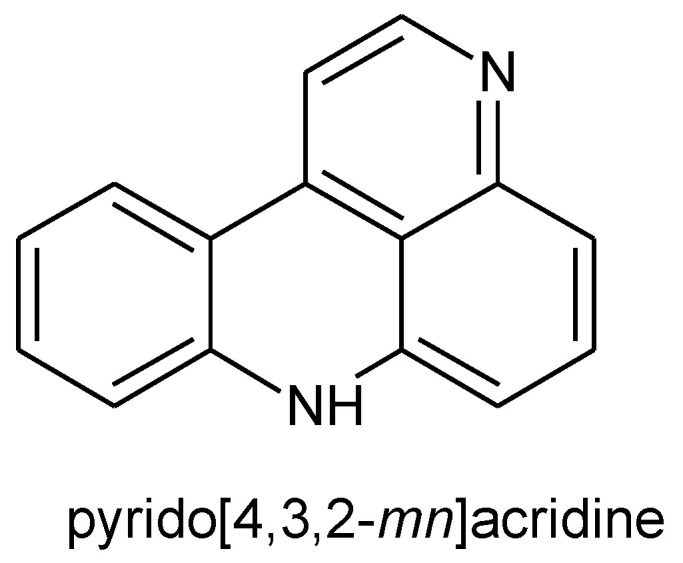
Structure of pyrido[4,3,2-*mn*]acridine.

Pyridoacridines are polyheterocyclic compounds containing pyrido[4,3,2-*mn*]acridine skeleton (Figure 30) and are a large number of marine-derived alkaloids.

Pyridoacridine alkaloids possess 2,7-naphthyridine scaffold in their structures and can be classified into tetracyclic, pentacyclic, hexacyclic, heptacyclic, and octacyclic compounds due to the number of rings attached to the pyrido[4,3,2-*mn*]acridine skeleton.

**Figure 31:**
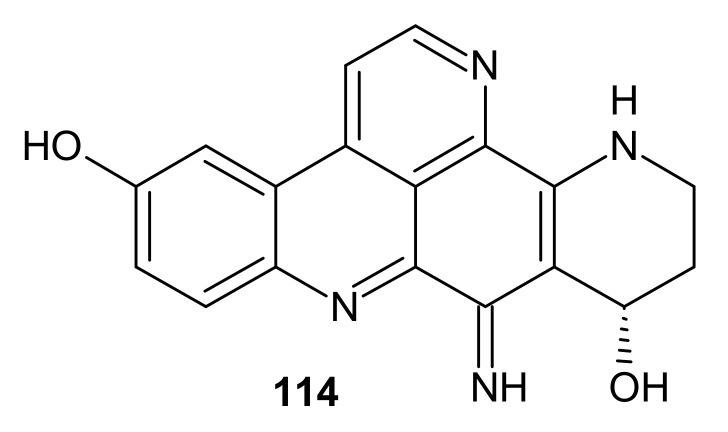
Calliactine 114.

Calliactine **114** (Figure 31) was the first pyridoacridine derivative that was obtained. This alkaloid **114** was isolated from the sea anemone *Calliactis parasitica* in 1940 by E. Lederer et al. [135].

Since then, about a 100 pyridoacridine analogs have been found in many marine organisms. They have been isolated from ascidians, sponges, anemone, and certain mollusks. Most pyridoacridine derivatives were reported to possess significant pharmacological activities, including anticancer, antimicrobial, and antiparasitic activities [136,137,138,139].

##### Tetracyclic Pyridoacridine Derivatives

**Figure 32:**
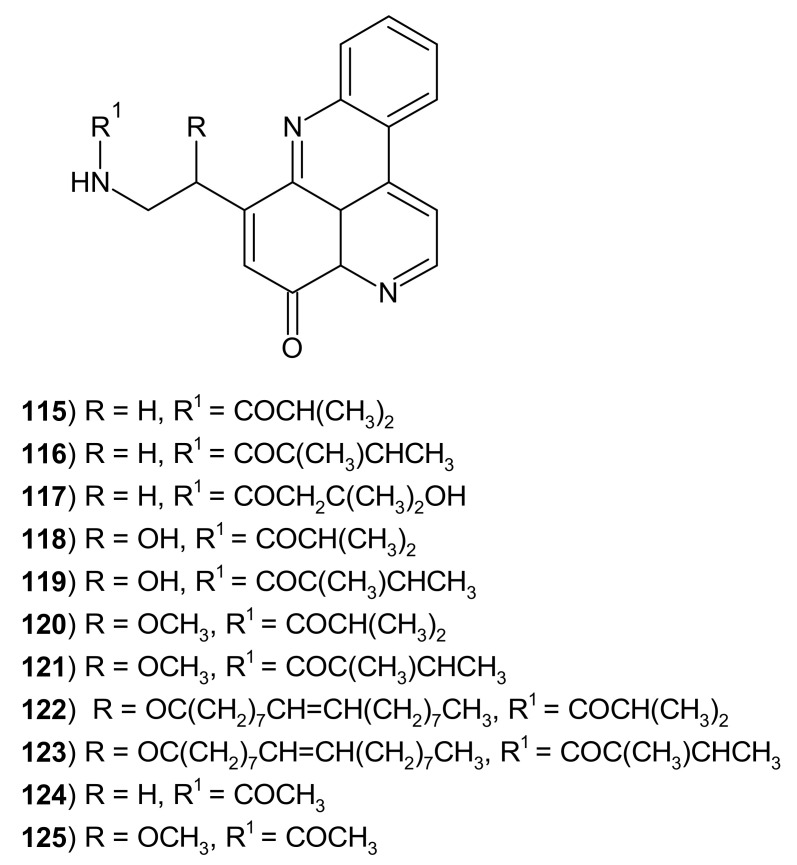
Cystodytines A–K **115**–**125**.

The first tetracyclic members of pyridoacridines were cystodytines **115**–**125** (Figure 32). These alkaloids **115**–**125** were isolated from the yellow tunicate *Cystodytes dellechiajei* by Kobayashi et al. [140,141]. Cystodytines A–K **115**–**125** were found to be cytotoxic. Cystodytine A–C **115**–**117** showed in vitro potent cytotoxicity against mouse leukemia cell lines with IC_50_ values of 0.22–0.24 μg/mL [140] and Cystodytines D–I **118**–**123** against murine lymphoma and human epidermoid carcinoma KB cells with IC_50_ values of 0.068–1.4 μg/mL [141]. Cystodytine J **124** isolated from *Cystodytes* sp. exhibited cytotoxic activity in vitro against the human colon tumor cell line with IC_50_ = 1.6 μM and inhibited the topoisomerase II with IC_50_ = 8.4 μM. The DNA binding ability of Cytodytine J **124** has also been reported [142]. 12-Methoxy derivatives **125** of cytodytine J were isolated from the ascidian *Lissoclinum notti*. Cystodytine K **125** showed cytotoxic activity in vitro against a murine leukemia cell line (IC_50_ = 1.3 μM) [143].

**Figure 33:**
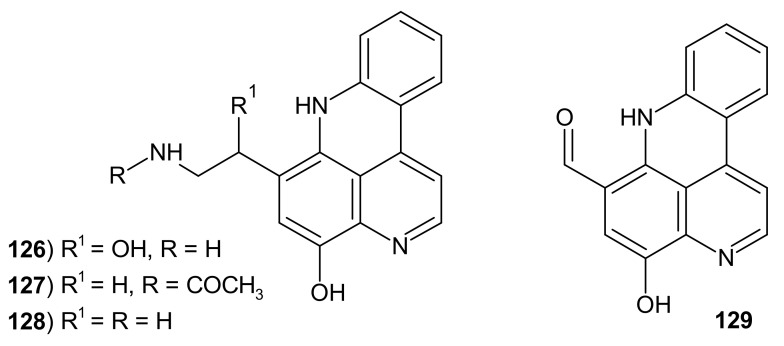
Styelsamines A–D **126**–**129**.

Styelsamines A–D **126**–**129** (Figure 33) were isolated from the ascidian *Eusynstyela latericius* [144]. Obtained alkaloids **126**–**129** showed cytotoxicity toward the human colon tumor cell line with IC_50_ values of 33, 89, 2.6, and 1.6 μM, respectively [144]. Styelsamines C **129** and D **128** were also isolated from the purple morph of the ascidian *Cystodytes dellechiajei* [145].

Fong and Copp [146] evaluated styelsamines **126**–**129** and cystodytines **115**–**125** for their DNA binding affinity and cytotoxic activity towards a panel of human tumor cell lines. Tested compounds showed moderate antiproliferative activity. Styelsamines B **127** and D **128** have particularly high affinity for calf thymus (CT)DNA, but cystodytines exhibited lower affinity [146].

**Figure 34:**
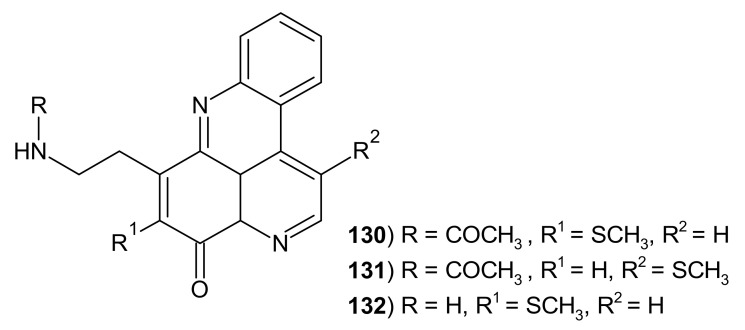
Diplamines 130–132.

Methylsulfanyl derivative of cystodytine J was isolated from the tunicate *Diplosoma* sp. by Charyulu et al. and named diplamine **130** [147]. Diplamine **130** and its isomer isodiplamine **131** (Figure 34) were also isolated from the ascidian *Lissoclinum notti* and were tested for their cytotoxicity against murine leukemia, human colon tumor, and non-malignant African Green Monkey kidney cell lines. Diplamine **130** was more cytotoxic towards BSC-1 cells than isodilamine **131**. Diplamine **130** turned out to be a stronger topoisomerase II inhibitor than etoposide and showed the ability to intercalate into DNA [142].

Diplamines **130**–**131** also exhibited moderate antimicrobial activity towards *Bacillus subtilis, Escherichia coli, Candida albicans,* and *Trichophyton mentagrophytes* [143]. Diplamine B **132** was isolated from the ascidian *Lissoclinum badium* and tested by immunoblotting for its effects on cellular p53 and Hdm2 in the tert-immortalized human retinal pigment epithelial cells (the potency was similar to proteasome inhibitor *N*-acetyl-leucyl-leucyl-norleucinal) [148].

The dependence of the activity of diplamine derivatives **130**–**132** on the type of substituents in the pyridoacridine scaffold is presented in Table 2.

**Figure 35:**
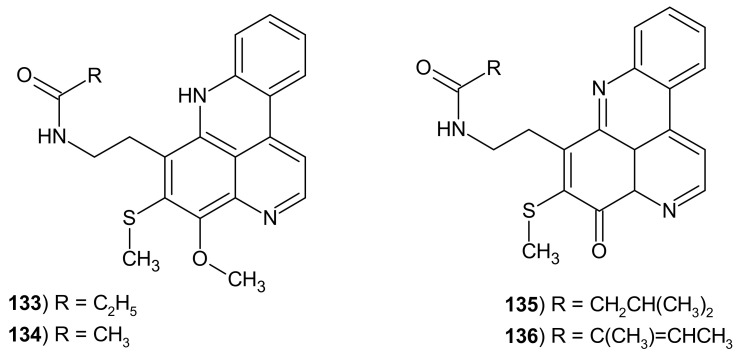
Veramines **133**, **134** and lissoclins **135** and **136**.

Another pyridoacridine alkaloid containing thiomethyl substituents—varamine A **133,** veramine B **134,** lissoclin A **135,** and lissoclin B **136** (Figure 35)—was isolated from the ascidian *Lissoclinum* sp. [149,150]. Varamines **133**–**134** showed cytotoxicity towards L-1210 murine leukemia cells with IC_50_ values of 0.03 and 0.05 μg/mL, so they proved to be more toxic than cystoditines, which have the same skeleton but without the thiomethyl group [150].

**Figure 36:**
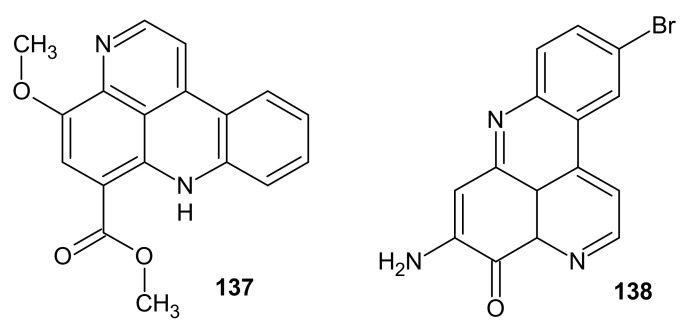
Norsegoline **137** and Pantherinine **138**.

Norsegoline **137** (Figure 36) was isolated from tunicate *Eudistoma* sp. [151,152]. Einat et al. [153] evaluated the inhibitory effect of norsegoline on the growth of myeloid progenitors obtained from bone marrow and peripheral blood of chronic myelogenous leukemia (CML) patients. Norsegoline **137** showed antiproliferative activity and may be an effective agent for use in removing ex vivo Philadelphia-positive cells from peripheral blood of CML patients in conjunction with autologous bone marrow transplantation [153].

Kim et al. [154] isolated a brominated alkaloid pantherinine **138** (Figure 36) from the ascidian *Aplidium pantherinum*, which showed cytotoxic activity against murine leukemia cells (ED_50_ = 4.5 µg/mL).

##### Penta- and Hexacyclic Pyridoacridine Derivatives

**Figure 37:**
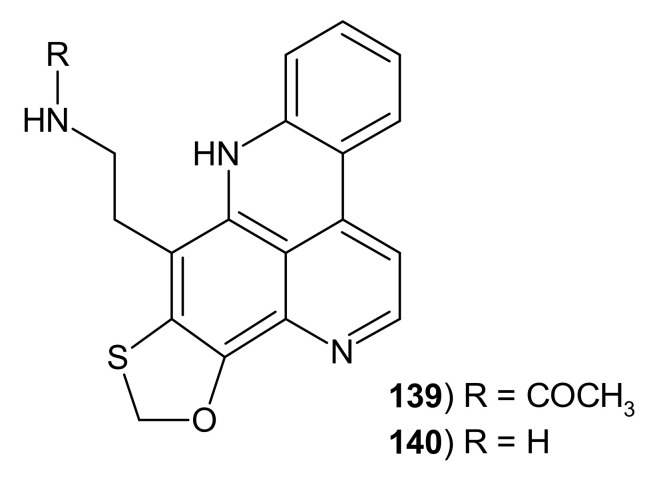
Lissoclinidines **139**–**140**.

Lissoclinidine **139** (Figure 37) was isolated from the ascidian *Lissoclinum notti* [143]. This pentacyclic alkaloid **139** is a product of diplamine photoreduction, where the thiomethyl group is cyclised into a 1,3-oxathiolane ring. Lissoclinidine **139** showed moderate antiproliferative activity [143].

Deacetyl derivative, lissoclinidine B **140,** was isolated from *Lissoclinum* cf. *badium* [155]. Lissoclinidine B **140** selectively induces cell apoptosis in a p53-dependent manner with IC_50_ values of 98.1 ± 6 μM and a dose-dependent increase in luciferase activity. The results of the studies showed that lissoclinidin B **140** is an inhibitor of Hdm2 auto-ubiquitylation and stabilizes p53 and Hdm2 in cells [148].

**Figure 38:**
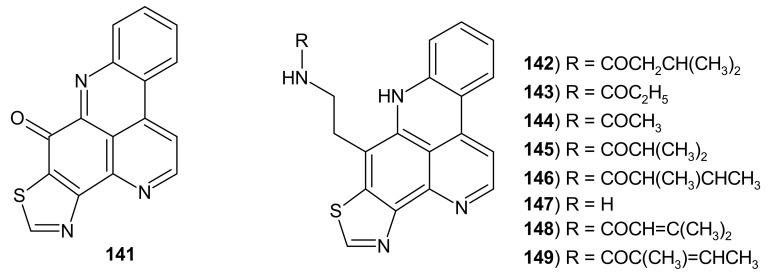
Kuanoniamines 141–149.

Kuanoniamine alkaloids **141**–**149** contain a thiazole ring fused to pyridoacridine scaffold (Figure 38). Kuanoniamines A–D **141**–**144** were first isolated from the mollusk *Chelynotus semperi* [156], and kuanoniamines E–F **145**–**146** were isolated by Nilar et al. [100] from Singaporean ascidians. *N*-Deacetylkuanoniamine C **147** was isolated from the Micronesian sponge *Oceanapia sp* [157]. Dehydrokuanoniamine B **148** and F **149** were isolated from South-Pacific Ocean ascidian *Cystodytes violatinctus* [158].

Cytotoxicity of kuanoniamines C **143** and D **144**, and *N*-deacetylkuanoniamine C **147** were studied in vitro, using two human cell lines (HeLa cells and MONO-MAC 6 cells) and exhibited similar activity with IC_50_ values of 1.2–2.0 μg/mL [157].

Kuanoniamine D **144** exhibited affinity to A1- and A2A-adenosine receptors (K_i_ values of 2.94 and 13.7 μM, respectively), and all derivatives showed moderate affinity to benzodiazepine binding sites of GABA-A receptors [157]. Dehydrokuanoniamine F **149** showed cytotoxic activity toward the SW480 colon cancer cell line with IC_50_ values of 3.30 μM [158].

**Figure 39:**
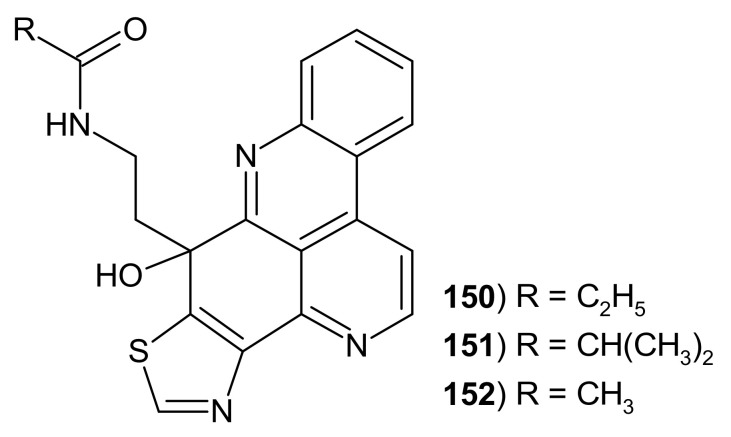
Sagitols 150–152.

Sagitols are hydroxy analogs of kuanoniamines (Figure 39). Sagitol **150** and sagitol C **151** were isolated from the Indonesian sponge *Oceanapia* sp. [159,160]. Sagitol C **151** exhibited antiproliferative activity towards mouse lymphoma, human cervix carcinoma, and rats brain tumor cell lines in MTT (the microculture tetrazolium) assay [161]. Sagitol D **152** was isolated from Vietnamese ascidians and showed a weak antioxidant activity with IC_50_ values of 92 μM in the DPPH (2,2-diphenyl-1-picryl-hydrazyl-hydrate) assay [160].

**Figure 40:**
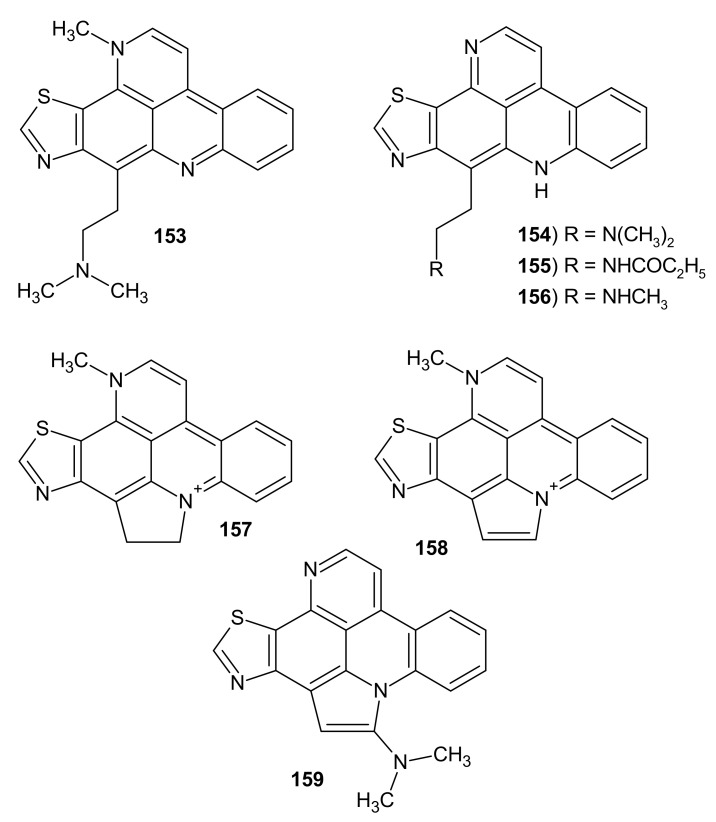
Dercitin analogs **153**–**159**.

Other pyridoacridine alkaloids containing a thiazole ring fused with pyridoacridine skeleton are Dercitin **153** isolated from *Dercitus* sp. sponges and its analogs isolated from *Stelletta* sp. sponges (Figure 40) [162,163,164]. Dercitin **153** exhibited in vivo antitumor activity. This alkaloid **153** inhibited the proliferation of murine leukemia and human leukemia, and lung, melanoma, and colon tumor cells with IC_50_ values of 63–150 nM. Dercitin **153** was a potent inhibitor of DNA polymerase I and showed an effect on the stabilization of protein–DNA complexes [165]. Dercitine analogs: nordecitine **154**, dercitamide **155**, dercitamine **156**, cyclodercitine **157**, dehydrocyclodercitine **158**, and stellettamine **159** also showed antiproliferative activity, but were less potent than dercitine **153** (Table 3) [166].

**Figure 41:**
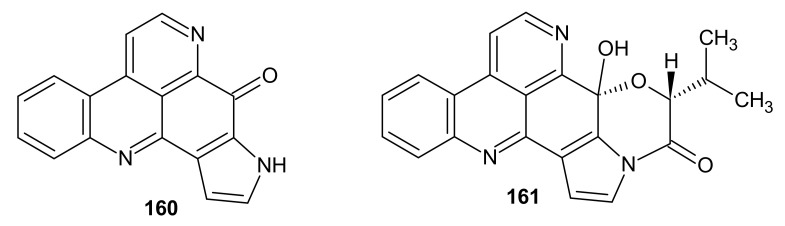
Sebastianine A **160** and B **161**.

Sebastianine A **160** has a pyrrole ring and sebastianine B **161** has a pyrrolidine ring fused with the pyridoacridine scaffold (Figure 41). These alkaloids **160**–**161** were isolated from the ascidian *Cystodytes dellechiajei* and showed cytotoxic activity against the HCT-116 colon carcinoma cells, indicating a p53-dependent mechanism [167].

**Figure 42:**
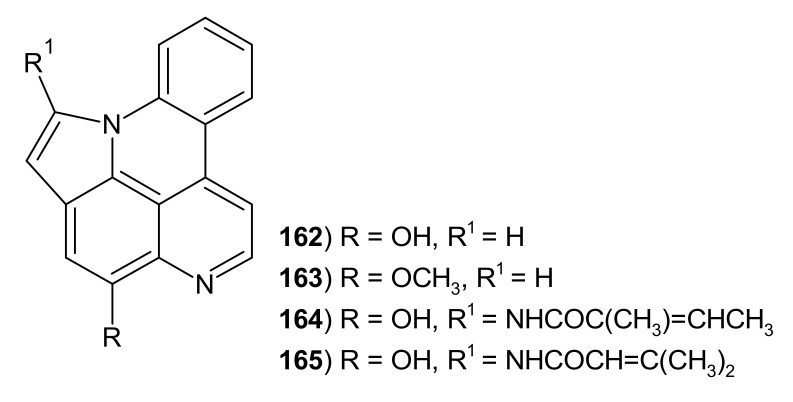
Arnoamines 162–165.

Arnoamines **162**–**165** have a pyrrole ring fused to the pyridoacridine scaffold (Figure 42). These cytotoxic alkaloids **162**–**165** were isolated from the ascidian *Cystodytes* sp. [158,168]. Arnoamine A **162** showed good antitumor activity against breast cancer cell lines with GI_50_ value of 0.3 μg/mL and weak activity against lung and colon cell lines with GI_50_ of 2.0 and 4.0 μg/mL, respectively. Arnoamine B **163** exhibited weak antiproliferative activity against the same cancer cell lines (GI_50_ of 2.0–3.0 μg/mL) [168]. Cytotoxic activity of arnoamine C **164** and arnoamine D **165** were evaluated against melanoma and colon cancer cell lines. Arnoamine D **165** was found to be more active than arnoamine C **164** (with IC_50_ values of 4.32–8.48 μM) [158].

**Figure 43:**
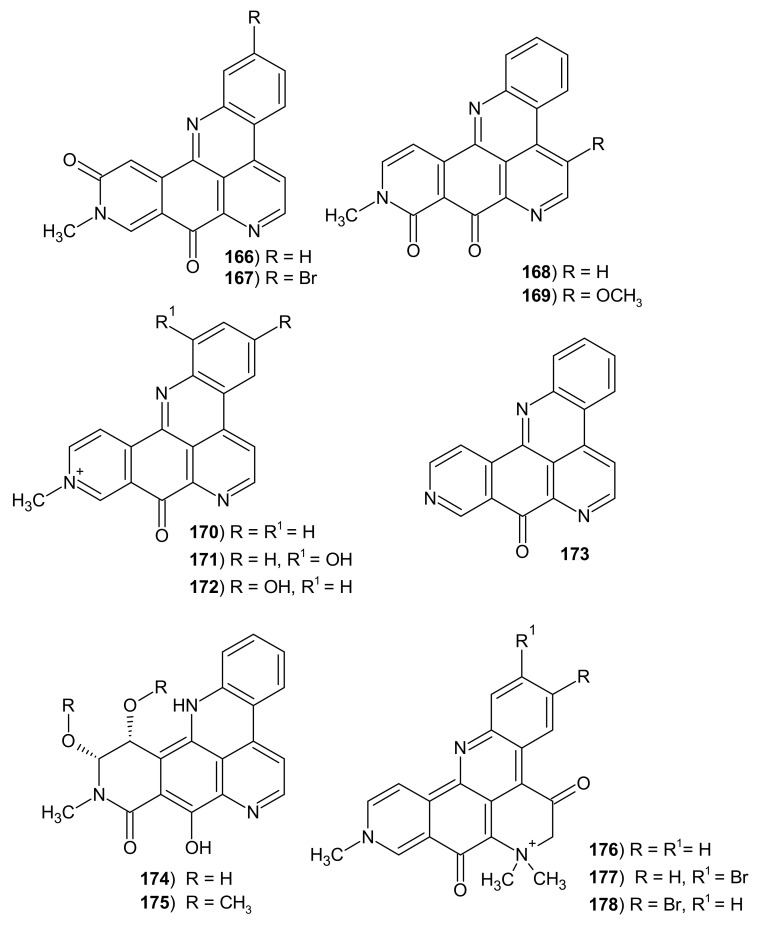
Alkaloids 166–178.

The first analog of pyridoacridine obtained from marine organisms was amphimedine **166**. This alkaloid **166** containing a pyridoquinoline skeleton was isolated from an *Amphimedon* sp. sponge [169]. Later, more amphinedine-type alkaloids (Figure 43) **166**–**178** were isolated from the marine sponge *Xestospongia* sp. and the ascidian *Cystodytes dellechiajei* [145,155].

Amphimedine analogs: amphimedine **166**, neoamphimedine **168**, deoxyamphimedine **170**, 1-hydroxy-deoxyamphimedine **171**, 3-hydroxy-deoxyamphimedine **172**, and debromopetrosamine **176** were evaluated in a zebrafish phenotype-based assay and only amphimedine **166** caused embryo necrosis, pericardial edema, and an enlarged yolk with a thin extension at 30 μM [170]. Amphimedine **166** exhibited cytotoxic activity toward P388 murine leukemia cells, but it did not inhibit topoisomerase II [169,171,172]. Neoamphimedine **168** was cytotoxic toward normal CHOAA8 cells and deoxyamphemidine **170** against human colon tumor cells [173]. Neoamphimedine **168** also showed antitrypanosomal activity against *T. brucei* with IC_50_ = 0.21 μM, but amphimedine **166** was inactive [171,173]. Demethyldeoxyamphimedine **173** showed antibacterial activity against *L. anguillarum* and *M. luteus* [145].

The presence of a bromine atom on the benzene ring determines the activity of these derivatives (Table 4). Petrosamine **177** isolated from *Petrosia* sp. sponge was found to be about six times more potent an AChE inhibitor than galanthamine (IC_50_ = 0.091 μM) [174]. Petrosamine B **178** weakly inhibited the *Helicobacter pylori* aspartyl semialdehyde dehydrogenase (IC_50_ = 306 μM) [175].

**Figure 44:**
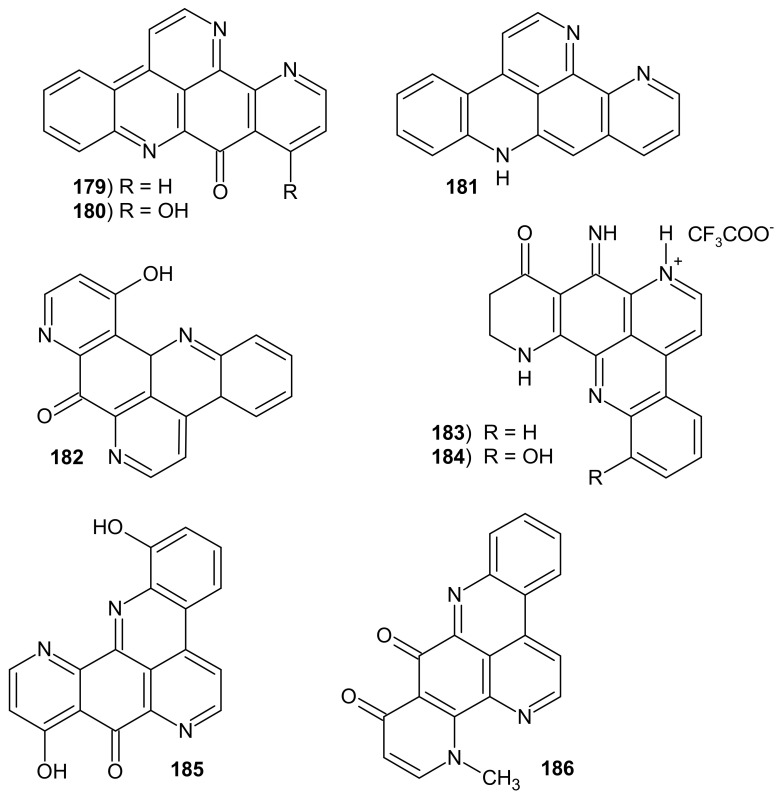
Structures of ascididemin analogs **179**–**186**.

Ascididemin analogs also possess a pyridine ring fused to the pyridoacridine scaffold (Figure 44). Ascididemin **179** was isolated from the tunicate *Didemnum* sp. and the ascidian *Cystodytes dellechiajei* [155], and later with 12-deoxyascididemin **181** from the ascidian *Polysyncraton echinatum* [176]. These alkaloids **179** and **181** showed potent activity against *T. brucei* with IC_50_ values of 0.077 and 0.032 μM, respectively [176]. Ascididemin **179** also exhibited antimicrobial activity against *C. resinae, E. coli*, and *B. subtilis* [177], and very good potency against *M. tuberculosis* (MIC = 0.35 μM) [178]. Ascididemin **179** causes release of calcium ions in the sarcoplasmic reticulum seven times more than caffeine [140]. Ascididemine analogs showed cytotoxic activities. Ascididemin **179** and 12-deoxyascididemin **181** exhibited cytotoxic activity toward the human embryonic kidney cell line with IC_50_ values of 1.48 and 7.63 μM, respectively [176]. Ascididemin **179** showed cytotoxic effects against murine leukemia cells (IC_50_ = 0.39 μg/mL) [140]. 11-Hydroxyascididemin **180** showed cytotoxic activity against the human prostate cancer (PC3) cell line with IC_50_ = 1.9 μM [179].

Meridine **182** (Figure 44) isolated from the ascidian *Amphicarpa meridiana* [180] and from the marine sponge *Ecionemia geodides* [181] also showed cytotoxic activity against the invasive bladder cancer cell lines (IC_50_ values of 3.76–4.56 μM) [181].

Ecionines A **183** and B **184** (Figure 44) possessing an imine moiety were isolated from *Ecionemia geodides* sponge [181]. Ecionine A **183** showed moderate cytotoxic activity against a panel of human bladder cancer cell lines with IC_50_ values of 3–7 μM [181].

Ancorine **185** and cnemidine A **186** are analogs of hydroxyascididemines (Figure 44). Ancorine **185** was isolated from the sponge *Ancorina geodides,* and cnemidine A **186** was isolated from the tunicate *Cnemidocarpa stolonifera* [182]. Cnemidine A **186** selectively inhibited PC3 with IC_50_ = 1.1 μM [182].

**Figure 45:**
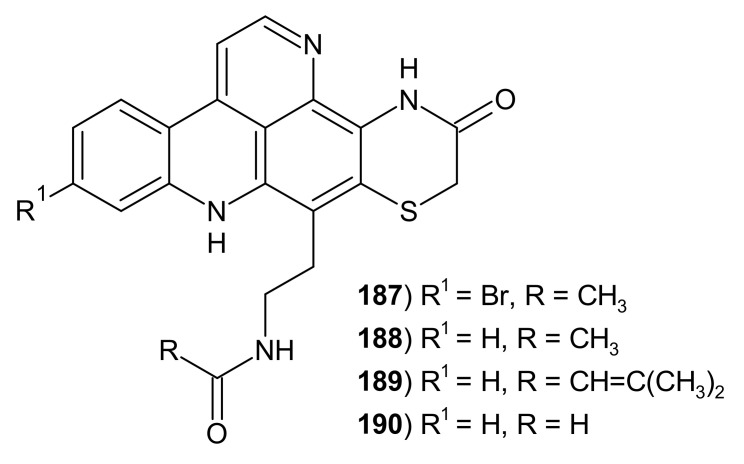
Shermilamines 187–190.

Shermilamines are alkaloids with the 3-thiomorpholinone ring fused to pyridoacridine scaffold (Figure 45). These alkaloids **187**–**190** were first isolated from the tunicate *Trididemnum* sp. [183,184], and later also from the ascidian *Cystodytes* sp. [142,185]. Shermilamines A **187** and B **188** exhibited cytotoxicity to murine leukemia cells. Shermilamine B **188** showed in vitro cytotoxic activity against KB cells with IC_50_ = 5 μg/mL, and human colon tumor cells with IC_50_ = 13.8 μM [156]. Shermilamines B and C **188**–**189** inhibit topoisomerase II and have the ability to intercalate into calf thymus DNA [142]. Shermilamine B **188** and *N*-deacetylshermilamine **190** were evaluated for their antibacterial activity against *E. coli* and *M. luteus*, but they were found to be less potent than reference gentamicin [138].

##### Hepta- and Oxacyclic Pyridoacridine Derivatives

**Figure 46:**
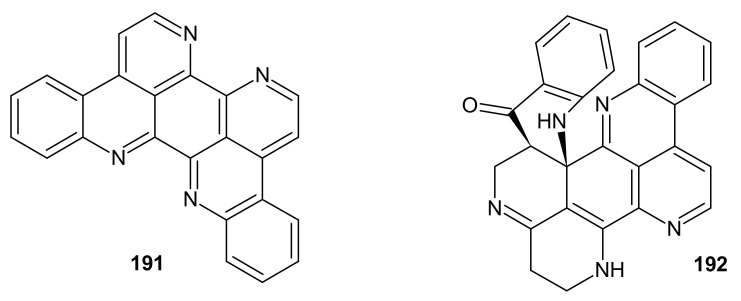
Eilatin **191** and biemnadin **192**.

Eilatin **191** is a symmetrical, heptacyclic alkaloid containing two pyridoacridine moieties (Figure 46). This alkaloid **191** was isolated from the tunicate *Eudistoma* sp. [151,152] and also from the ascidians *Cystodytes* sp. and *Polysyncraton echinatum* [142,169]. Eilatin **191** showed in vitro antiproliferative activity against the human colon tumor cell line and the human embryonic kidney cell line [176]. It also inhibited topoisomerase II and intercalated into DNA [142]. Eilatin **191** has two sets of nitrogen atoms capable of metal ion chelation. Complexes of eilatin–Ru(II) exhibited strong anti-HIV activity [186].

Biemnadin **192** is the octacyclic alkaloid containing the 2,7-naphthyridine ring in its skeleton (Figure 46). This alkaloid **192** was isolated from *Biemna* sp. sponges [187]. Biemnadin **192** showed weak cytotoxic activity toward the superficial bladder cancer cell line [181] and induced multipolar neuritogenesis [188].

### 2.6. Naphthyridines Molecular Mechanisms of Action—What Do We Know?

Naturally occurring naphthyridines are characterized by diverse mechanisms of action. Some of their biomolecular activities have been discovered, nevertheless there is still much to explore in this field.

Naphthyridines obtained from natural sources are characterized by anticancer potency. The compounds exhibit features of topoisomerase inhibitors and DNA intercalators [142]. They were documented to induce apoptosis in cancer cells in both p53-dependent and p53-independent manner [23,25,148,167]. Moreover, the compounds interfere with procytotoxic signaling pathways, i.a., AKT/mTOR, ERK, JNK, WNT [38,39,40,62]. Antineoplasm properties against drug-resistant tumors could be elucidated by inhibiting efflux-pumps activity, however, this topic requires further study [41,42].

Anti-inflammatory activity of naphthyridines is based on reducing iNOS and COX-2 release, decrease in NO production, and IL-6 and TNF-α secretion [7,15,54,56,57]. They suppress autoimmunity reactions through an IFN-β/IL-27/IL-10 pathway, and by targeting proautoimmunity gene expression including angiogenin and stratifin [48,49].

The compounds modulate neurotransmission by affinity to GABA A and MAO A receptors, and inhibiting AchE activity [29,157,174].

Natural naphthyridines antiinfectious properties were widely screened against many infectious species [6,8,111,112,178]. However, molecular mechanisms of their action are still poorly researched and described. This issue is an interesting target for deeper research.

Natural naphthyridines have an extensive background of preliminary research, which is a solid foundation for molecular studies. Further investigations are still needed.

## 3. Conclusions

The natural environment, including marine and terrestrial organisms, should be considered as a rich source of bioactive substances. Naturally-derived naphthyridines, isolated mostly from sea species and terrestrial plants, have been shown as potent chemical compounds with multidirectional activity. Natural naphthyridines are most abundantly represented by 1,6- and 2,7-naphthyridine isomeric forms. Until now, research studies revealed their impressive antimicrobial [6,8,177], antifungal [6,8,111,112], and antimycobacterial [10,178] effects, and some of them displayed activity superior or comparable to those presented by chloramphenicol [112], amphotericin B [115], and rifampin [124]—drugs used in standard therapeutic regimens. Moreover, naphthyridines were shown to present significant properties including antiinfectious: antiviral [11,24,50,51], antiparasitic [9,176], and antimalarial [113]; anticancer [5,6,23,35]; influencing cardiovascular system: hypotensive [16,27], cardioprotective [43,44]; neurological: sedative [94], analgesic [15], anticonvulsant [78], stimulating neuritogenesis [188]; psychotropic: antianxiety [97], antidepressant [29,97]; and affecting immune system: anti-inflammatory [7,56,57], immunosuppressant [48,49,50]. The spectrum of activity of naturally-derived naphthyridines is wide, thus these compounds are undeniably fascinating subjects of research. An undoubted advantage of naphthyridines is their wide availability thanks to the possibility of obtaining them both from natural sources and synthetically. The versatility of naphthyridines is expressed by the occurrence of multiple activities within a single compound. Moreover, many of the representatives are considered as safe and nontoxic, and constitute a great alternatives for standard therapies [9,48]. We strongly believe that this work will contribute to further exploration of naphthyridine derivatives—their natural sources and bioactive properties—and will result in the use of these chemical compounds for therapeutic purposes in the future.

## Figures and Tables

**Table 1:** The dependence of the activity of alkaloids **92**–**96** on the types of substituents.

Activity	Substituents			
	R	R^1^	R^2^	R^3^
anticancer	H	H, OCH_3_	H, OCH_3_	H, OCH_3_
antimalarial	H, CH_3_	H	H	H
antifungal	H, CH_3_	H	H	H
antibacterial	H, CH_3_	H	H	H

**Table 2:** The dependence of the activity of alkaloids **130**–**132** on the type of substituents.

Activity	Substituent		
	R	R^1^	R^2^
anticancer	H, COCH_3_	SCH_3_	H
antimicrobial	COCH_3_	H, SCH_3_	H, SCH_3_

**Table 3:** Antiproliferative activity of dercitine analogs **153**–**159**.

Alkaloid	Activity	IC_50_
**153**	antiproliferative inhibition of polymerase I stabilization of protein-DNA complexes	63–150 nM
**154**	antiproliferative	4.8 μM
**155**	antiproliferative	26.7 μM
**156**	antiproliferative	12.0 μM
**157**	antiproliferative	1.9 μM
**158**	antiproliferative	9.9 μM
**159**	antiproliferative	60.0 μM

**Table 4:** The dependence of the activity of petrosamine analogs **176**–**178** on the type of substituents.

Activity	Substituent	
	R	R^1^
AChE inhibitor	H	Br
antibacterial (*H. pylori*)	Br	H
inactive	H	H
